# Varying mechanical forces drive sensory epithelium formation

**DOI:** 10.1126/sciadv.adf2664

**Published:** 2023-11-03

**Authors:** Mingyu Xia, Mingxuan Wu, Yuanrong Li, Yaoqian Liu, Gaogan Jia, Yiyun Lou, Jiaoyao Ma, Qing Gao, Mingjun Xie, Yuewei Chen, Yong He, Huawei Li, Wenyan Li

**Affiliations:** ^1^ ENT institute and Otorhinolaryngology Department of Eye & ENT Hospital, State Key Laboratory of Medical Neurobiology and MOE Frontiers Center for Brain Science, Fudan University, Shanghai 200031, China.; ^2^ Institutes of Biomedical Sciences, Fudan University, Shanghai 200032, China.; ^3^ NHC Key Laboratory of Hearing Medicine, Fudan University, Shanghai 200031, China.; ^4^ The Institutes of Brain Science and the Collaborative Innovation Center for Brain Science, Fudan University, Shanghai 200032, China.; ^5^ State Key Laboratory of Fluid Power and Mechatronic Systems, School of Mechanical Engineering, Zhejiang University, Hangzhou 310027, China.; ^6^ Key Laboratory of 3D Printing Process and Equipment of Zhejiang Province, School of Mechanical Engineering, Zhejiang University, Hangzhou 310027, China.; ^7^ Cancer Center, Zhejiang University, Hangzhou, Zhejiang 310058, China.; ^8^ Plastic and Reconstructive Surgery Center, Department of Plastic and Reconstructive Surgery, Zhejiang Provincial People’s Hospital, Affiliated People’s Hospital, Hangzhou Medical College, Hangzhou, Zhejiang 310014, China.; ^9^ Shanghai Engineering Research Centre of Cochlear Implant, Shanghai 200031, China.

## Abstract

The mechanical cues of the external microenvironment have been recognized as essential clues driving cell behavior. Although intracellular signals modulating cell fate during sensory epithelium development is well understood, the driving force of sensory epithelium formation remains elusive. Here, we manufactured a hybrid hydrogel with tunable mechanical properties for the cochlear organoids culture and revealed that the extracellular matrix (ECM) drives sensory epithelium formation through shifting stiffness in a stage-dependent pattern. As the driving force, moderate ECM stiffness activated the expansion of cochlear progenitor cell (CPC)–derived epithelial organoids by modulating the integrin α3 (ITGA3)/F-actin cytoskeleton/YAP signaling. Higher stiffness induced the transition of CPCs into sensory hair cells (HCs) through increasing the intracellular Ca^2+^ signaling mediated by PIEZO2 and then activating KLF2 to accomplish the cell specification . Our results identify the molecular mechanism of sensory epithelium formation guided by ECM mechanical force and contribute to developing therapeutic approaches for HC regeneration.

## Teaser

ECM stiffness serves as the driving force of inner ear sensory epithelium formation in a stage-dependent manner.

## INTRODUCTION

Sensory organs responsible for hearing, vision, smell, taste, and touch are essential for perceiving and navigating the world. The ectoderm is the common origin of diverse sensory organs, which further develop into unique and complex structures over time (*1*, *2*). The formation of respective sensory regions comprises two major events: the expansion of a shared pool of progenitor cells, followed by the specification of the progenitor cells into various cell types containing sensory cells (*3*). Although the discovery of multiple genes and signaling pathways has led to our understanding of the vital role of intracellular signals during development (*4*–*6*), the original driving force of the sensory epithelium formation remains largely uncovered.

Among these sensory organs, the hearing sensory organ is one of the most complex structures in mammals. It originates from a short-bent tube in the inner ear and then further extends and coils to form the three-dimensional (3D) spiral shape, within which hair cells (HCs) and supporting cells (SCs) differentiated from the shared pool of progenitors and become prototypically arranged in mosaic patterns (*4*). Highly specialized HCs are essential for hearing perception by converting mechanical stimulation from external sound waves into electrochemical signals. Apart from the intracellular signaling cascades triggered by diffusible factors from the microenvironment (*7*) and by intercellular lateral inhibition from neighboring cells (*8*), the mechanical forces generated by the extracellular matrix (ECM) have gradually been recognized as another essential factor driving organ development. For example, a recent study suggested that hyaluronate (HA) pressure is the driving force for semicircular canal morphogenesis, which means an essential role for ECM mechanical force in shaping buds into tubes during inner ear development (*9*). It has also been reported that mechanical forces contribute to shaping the arrangement of the sensory epithelium of the cochlea (*8*), and the developing HCs and SCs have different mechanical properties on the tissue surface during inner ear development (*10*). Although mechanical forces have been highlighted in the cellular arrangement and morphological transformation of the sensory epithelium, the origin of mechanical cues and their effects on cell fate switch are not well understood.

The relation between ECM mechanical forces and sensory epithelium generation remains poorly understood because of the inaccessibility of the inner ear. Over the past decade, organoids generated from stem cells or progenitor cells have been shown to recapitulate multiple aspects of corresponding organs, making them a promising model for studying organ development (*11*, *12*). Furthermore, the defined 3D matrix for organoid culture can mimic the microenvironment in vivo by providing spatial support, ECM components, and mechanical pressure, which make organoids ideal models for dissecting the effects of single or multiple factors on organogenesis. Recently, we have described that ECM cues from Matrigel promote the proliferation of inner ear progenitor cells via the RhoA-YAP-β-catenin mechanotransduction axis, which provides the first piece of evidence that the mechanical forces might influence the inner ear development and HC regeneration (*13*). Yet, Matrigel is a heterogeneous hydrogel extracted from Engelbreth-Holm-Swarm mouse sarcoma, which features complex, variable compositions and has risks of immunogenicity or tumorigenicity (*14*). Matrigel is a soft matrix with poor controllability on physical and biochemical properties, which brings limitations for investigating the effects of ECM mechanical cues on the morphogenesis of cochlear progenitor cell (CPC)–generated organoids. To dissect the roles of mechanical cues in guiding sensory epithelium morphogenesis, a controllable hydrogel system with high biocompatibility and defined mechanical property is urgently required.

Here, we constructed a stiffness-adjustable gelatin methacryloyl (GelMA)–HA-Arg-Gly-Asp (RGD) hydrogel system for the culture of cochlear organoids. By investigating the biological behaviors of CPCs in the designed mechanical environment of the hybrid hydrogel, we demonstrated that medium ECM stiffness stimulated CPC proliferation through a mechanism requiring ITGA3/F-actin cytoskeleton/YAP signaling. Increasing the stiffness suppressed the proliferation of CPCs and stimulated the differentiation toward HCs, which was transduced by the mechanically sensitive Ca^2+^/extracellular signal–regulated kinase 1/2 (ERK1/2)/Kruppel-like factor 2 (KLF2) signaling pathway. Our findings suggest that ECM mechanical forces were the driving force for triggering intracellular signal cascades to guide sensory epithelium generation.

## RESULTS

### The design of a mechanically tunable matrix system for the construction of cochlear organoid

**Fig. 1.**
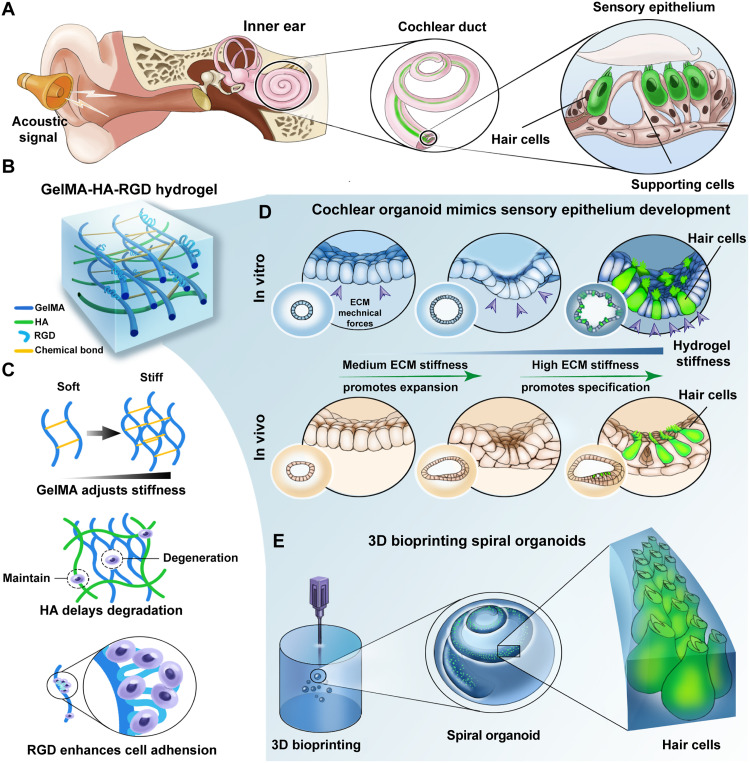
Stiffness-adjustable GelMA-RGD-HA hydrogels for cochlear organoid culture. (**A**) Schematic representation of HCs in the sensory epithelium of the inner ear for acoustic perception. (**B**) Synthesis and application of GelMA-RGD-HA hydrogels. (**C**) The role of each component of hydrogels. (**D**) Schematic depicting gradient stiffness from hydrogel modulating staged sensory epithelium formation of organoids, which mimic the process of sensory epithelium development in vivo. (**E**) 3D bioprinting spiral cochlear organoids based on hybrid hydrogels.

Sensory HC resided in the cochlear epithelium, which is responsible for the perception of sound (Fig. 1A). To explore whether ECM mechanical forces regulate sensory epithelium generation, we synthesized a defined matrix for cochlear organoid culture (Fig. 1B). Gelatin methacrylate served as the backbone of the hydrogel, which shows excellent tunable mechanical properties and rapid cross-linking ability (Fig. 1C). Hydrogel-based scaffolds were reinforced with HA to improve physical stability. RGD ligand, integrin-binding sequences for cell attachment, was designed to enhance biocompatibility by mediating the cell-matrix interaction.

By precisely tuning the stiffness of the hydrogel, cells in organoids showed staged expansion and specification into sensory HCs. In the stiffness-tunable hydrogel system, the mechanical force from hydrogels driving cochlear organoid formation mimicked the dynamic ECM force shaping sensory epithelium formation in vivo (Fig. 1D). In addition, we uncovered two independent mechanotransduction mechanisms that could respond to the varied ECM stiffness to promote CPC expansion and sensory HC generation. We further used 3D bioprinting with the hybrid hydrogel to generate spiral cochlear organoids with sensory HCs, which highly mimicked the whole structure of the sensory epithelium of postnatal cochlea (Fig. 1E).

### Characterization of the physicochemical properties of biocompatible GelMA-HA-RGD hydrogel

**Fig. 2.**
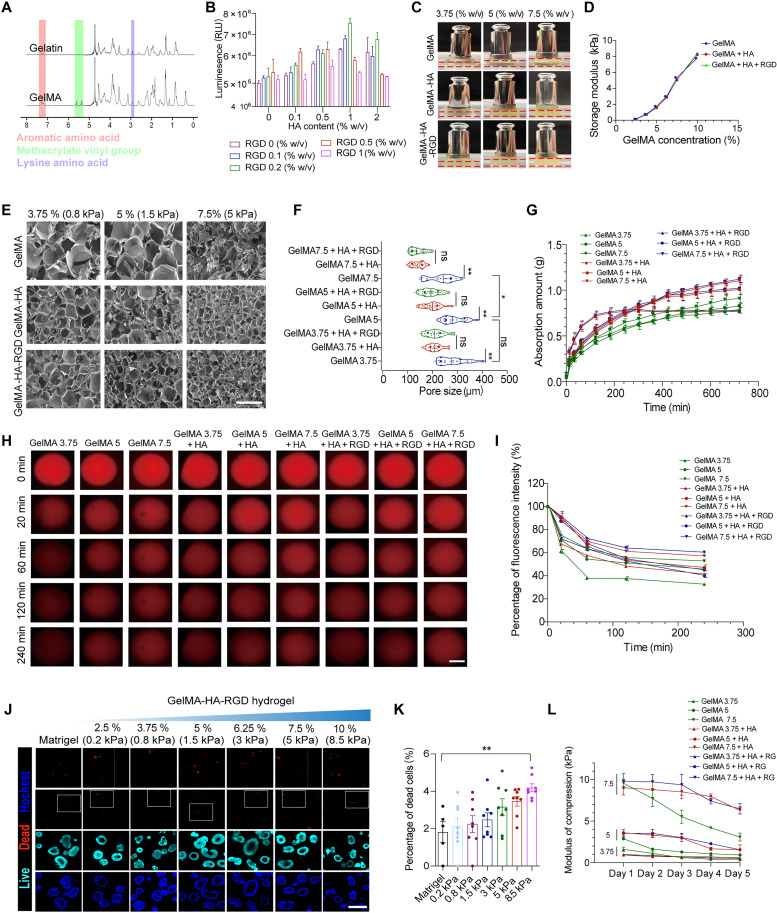
Physicochemical characterization of the GelMA-HA-RGD hydrogel. (**A**) H NMR spectra of unmodified gelatin and GelMA macromers. (**B**) Luminescent cell viability assay measuring organoids cultured in 5% (w/v) GelMA combined with different contents of HA and RGD. (**C**) Image showing hydrogel blocks with different materials deformed at different heights when pressed by the same weight. (**D**) The changes in the energy storage modulus of the indicated groups of the indicated hydrogels. (**E**) Scanning electron microscopy images of the indicated hydrogels. Scale bar, 600 μm. (**F**) Quantification of pore size of the indicated hydrogels. Eighteen to 32 pores of each group, three independent experiments. (**G**) The water absorption speed of dried hydrogel blocks of different compositions. (**H**) The confocal fluorescent microscopy images showing the release of rhodamine. Scale bar, 500 μm. (**I**) Percentage of fluorescence intensity from (H). (**J**) Live/dead staining of organoids cultured in Matrigel and GelMA-HA-RGD hydrogels with different stiffness. Scale bar, 200 μm. (**K**) The number of dead cells per organoid. Seven to eight organoids at each condition, three independent experiments. The data are presented as the means ± SEM, **P < 0.01 versus Matrigel. (**L**) Stiffness of the different material groups during cell culture. The data are presented as the means ± SEM, *P < 0.05, **P < 0.01; ns, not significant [one-way analysis of variance (ANOVA) followed by Tukey’s multiple comparisons test].

To develop a matrix with tunable mechanical properties for efficient organoid formation from CPCs, we established 3D hydrogel networks based on the combination of GelMA, HA, and RGD. The conjugation of methacryloyl groups to gelatin molecules was confirmed by the H nuclear magnetic resonance (NMR) spectra and 2,4,6-trinitrobenzene–sulfonic acid assay (Fig. 2A and fig. S1A). The degree of methacryloyl substitution of fabricated GelMA was about 60%, giving the GelMA a wide range of controllable stiffness. Single dissociated CPCs were then embedded into the 5% (w/v) GelMA gels cross-linked with different concentrations of HA and RGD to determine the appropriate combinations for CPC growth. After culturing for 7 days, cells in 1% (w/v) HA and 0.2% (w/v) RGD–modified GelMA hydrogel exhibited the most robust viability (Fig. 2B). We compressed the hybrid gels with different GelMA concentrations (3.75, 5, and 7.5%) for elementary evaluation. The different heights under the same weight compression indicated that the mechanical properties varied by GelMA concentration (Fig. 2C). We further performed Young’s modulus assay, and the GelMA concentration was positively correlated with the storage modulus, an essential Young’s modulus parameter, which suggested that increased GelMA concentration resulted in enhanced stiffness in the cross-linked hydrogels (Fig. 2D). The storage modulus of solidified Matrigel was also tested, which was quite close to hydrogel with 5% GelMA (fig. S1B). In the GelMA-HA-RGD hydrogels without cross-linking, both the storage and loss modulus of hydrogels decrease with different GelMA concentrations with increasing temperature (fig. S1C). Hydrogel with a higher proportion of GelMA renders the hydrogel with a stronger shape retention ability indicated by storage modulus data. To a great extent, GelMA-HA-RGD hydrogel presents power-law shear thinning behavior (viscosity decreases with increasing shear stress) (fig. S1D). At the same time, no substantive difference was found by cross-linking with 1% HA or 0.2% RGD. Scanning electron microscopy was used to analyze the structure in detail, and we found that the pore size of the gels decreased with increasing GelMA concentration and the addition of HA (Fig. 2, E and F).

To assess the gels’ water absorption capacity, the dried samples were placed in deionized water to measure the weight change. All of the gels had excellent water absorption capacity (fig. S1E), and a lower concentration of GelMA reached saturation more quickly, while a higher concentration of GelMA had greater water retention (Fig. 2G). In addition, HA increased the water absorption rate of the system, which might be due to each HA unit containing four hydroxyl groups (-OH). Hydrogel microspheres containing rhodamine were fabricated to measure the permeability of the hydrogels. As shown in Fig. 2H, the mean fluorescence levels of the rhodamine immersed in Dulbecco’s phosphate-buffered saline (DPBS) decreased over time, indicating that the rhodamine was released from the microspheres. The fluorescence intensity was reduced faster in the gels with lower concentrations of GelMA than in those with higher concentrations (Fig. 2, H and I). The addition of HA reduced the release rate of rhodamine from microspheres, indicating that HA increases the water retention capacity of the hydrogels.

The average proportion of dead cells within the CPC-derived organoids was 1.83% in Matrigel after 7 days of culture, and this increased gradually as the mechanical stiffness of the gel increased. Compared with Matrigel, the increase in the proportion of dead cells was significant only in the stiffest GelMA-HA-RGD gel at 8.5 kPa (average of 4.19% dead cells) (Fig. 2, J and K). These results showed that the hybrid GelMA-HA-RGD hydrogels provided an ideal stiffness-adjustable matrix for the culture of organoids from CPCs. We then compared the degradation rates of hydrogels laden with CPCs in different compositions and found that the addition of HA retarded the degradation rate of the gels (Fig. 2L), indicating that HA increases the mechanical stability of the hydrogel during culture.

### Hybrid hydrogel stiffness regulates CPC-derived organoid formation

**Fig. 3.**
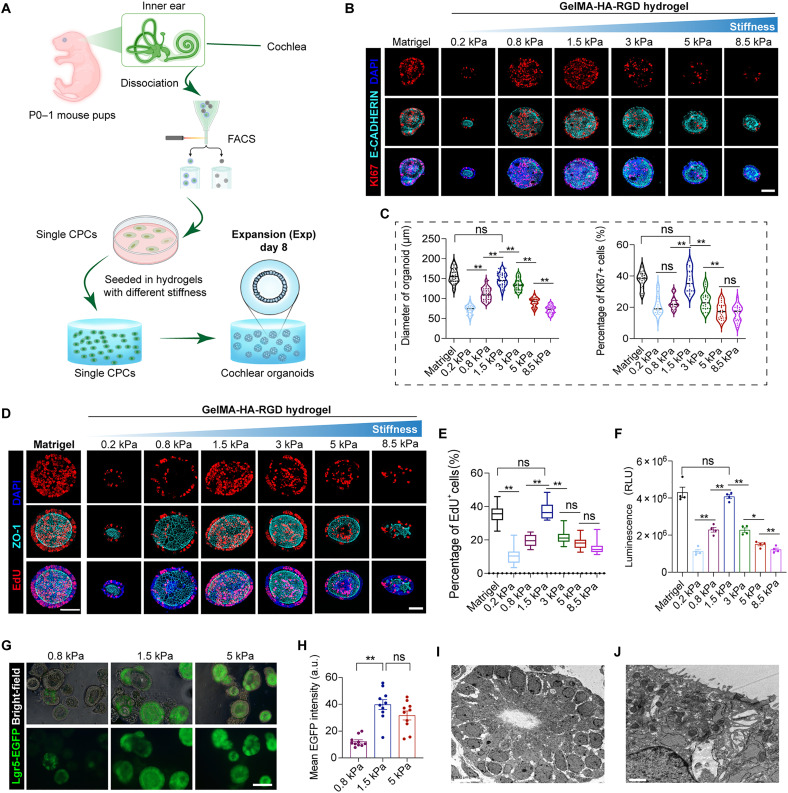
Medium matrix stiffness promotes cochlear organoid expansion. (**A**) Schematic depicting the experimental strategy for cochlear organoid expansion in hydrogels with different stiffness. (**B**) Confocal images show KI67 and E-CADHERIN staining of organoids cultured in Matrigel and hybrid hydrogels with different stiffness. Scale bar, 50 μm. (**C**) Quantifying the diameter of organoids and the percentage of KI67-positive cells per organoid from (B). Forty organoids (for diameter) and 20 organoids (for KI67) at each condition, three independent experiments. (**D**) Confocal images show EdU and ZO-1 staining of organoids cultured in Matrigel and hybrid hydrogels with different stiffness. Scale bar, 50 μm. (**E**) The percentage of EdU-positive cells per organoid from (D). Twenty organoids at each condition, three independent experiments. (**F**) Luminescent cell viability assay measuring CPCs cultured in Matrigel and different stiffness hydrogels (*n* = 3 independent experiments). (**G**) Bright-field and fluorescence microscopy images showing *Lgr5*-EGFP expression of organoids in hydrogels with different stiffness. Scale bar, 100 μm. (**H**) Three independent experiments are quantifying the EGFP intensity from (G). Ten organoids at each condition. a.u., arbitrary units. (**I** and **J**) Transmission electron microscopy images of cochlear organoids in 1.5-kPa hybrid hydrogel. The data are presented as the means ± SEM, *P < 0.05, **P < 0.01 (one-way ANOVA followed by Tukey’s multiple comparisons test).

Using the stiffness-tunable hydrogel system by adjusting GelMA concentration, we investigated sensory epithelium generation in organoids from CPCs under different stiffness ranging from 0.2 to 8.5 kPa (Fig. 3A). After 8 days of culture, organoids could be generated in the GelMA-HA-RGD hydrogel and Matrigel, which preserved the typical morphology of epithelial organoids and showed the intact expression of the epithelium markers E-CADHERIN and ZO-1 (Fig. 3, B and D). The size of the organoids gradually increased with the growing stiffness of hydrogel from 0.2 to 1.5 kPa, achieving a comparable size in 1.5-kPa hydrogel as in Matrigel, while further increasing the stiffness of the hydrogel led to the reduced size of organoids (Fig. 3, B and C). Moreover, KI67 and 5-ethynyl-2'-deoxyuridine (EdU) staining in organoids cultured in GelMA-HA-RGD confirmed the dynamic trend of the proliferative capacity of CPCs under different stiffnesses, and the proliferative rate in the 1.5-kPa gel was comparable with the Matrigel control (Fig. 3, B to E). The assessment of adenosine triphosphate levels further confirmed that CPCs cultured in 1.5-kPa gel exhibited the highest cell viability (Fig. 3F). In addition, *Lgr5*, a marker for CPCs, was highly expressed in the 1.5-kPa gel (Fig. 3, G and H). Transmission electron microscopy showed that organoids in the 1.5-kPa gel were composed of layered cells with tight junctions, and many microvilli facing toward the lumen were observed at the top of the epithelial cells (Fig. 3, I and J).

**Fig. 4.**
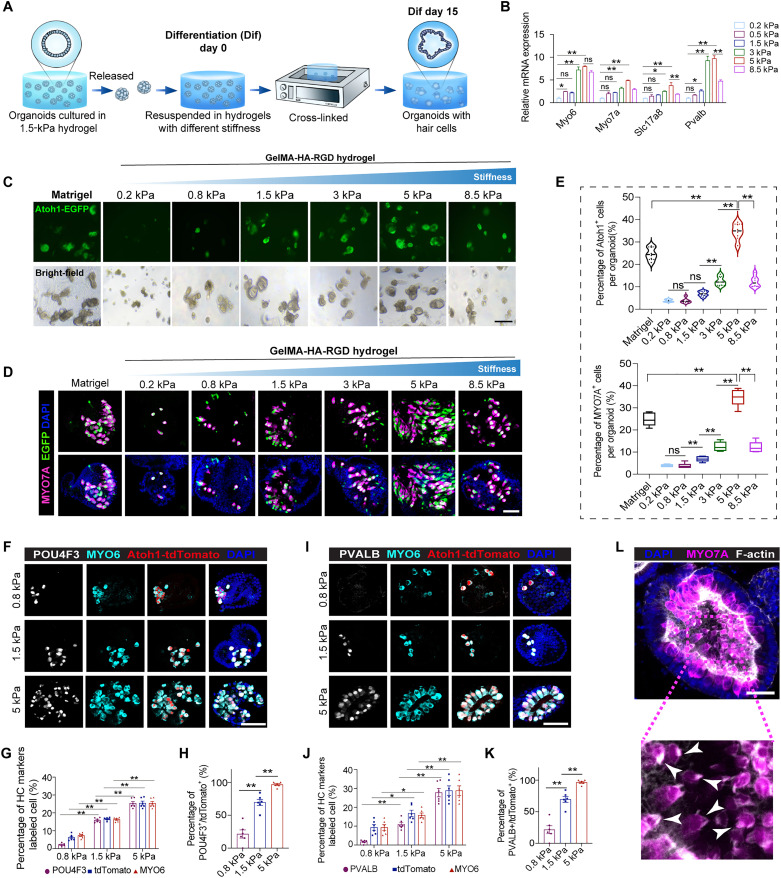
Higher matrix stiffness enables organoid specialization into HC organoids. (**A**) Schematic depicting the experimental strategy for cochlear organoid differentiation in hydrogels with different stiffness. (**B**) Real-time PCR analysis showing the relative expression of markers of HCs. The results were normalized to glyceraldehyde-3-phosphate dehydrogenase (*Gapdh*) in the same sample and then normalized to the control group (*n* = 3 independent experiments). (**C**) Fluorescence and bright-field microscopy images showing* Atoh1*-EGFP expression of organoids cultured in Matrigel and hybrid hydrogels with different stiffness. Scale bar, 250 μm. (**D**) Confocal images show MYO7A staining and *Atoh1*-EGFP in organoids cultured in Matrigel and hybrid hydrogels with different stiffness. Scale bar, 50 μm. (**E**) The percentage of *Atoh1*-EGFP^+^ and MYO7A^+^ cells per organoid from (D). Six organoids at each condition, three independent experiments. (**F**) Confocal images show *Atoh1*-tdTomato, MYO6, and POU4F3 staining in organoids cultured in hydrogels with different stiffness. Scale bar, 50 μm. (**G**) The percentage of indicated positive cells per organoid from (F). (**H**) The rate of POU4F3^+^/tdTomato^+^ cells per organoid from (F). (**I**) Confocal images show *Atoh1*-tdTomato MYO6 and PVALB staining of organoids cultured in hydrogels with different stiffness. Scale bar, 50 μm. (**J**) The percentage of indicated positive cells per organoid from (I). (**K**) Quantifying the rate of PVALB^+^/tdTomato^+^ cells per organoid from (I). Six organoids at each condition, three independent experiments. (**L**) Confocal images show MYO7A and F-actin staining of organoids cultured in stiff hybrid hydrogels. Scale bar, 25 μm. The arrowheads indicate HC bundles. The data are presented as means ± SEM, *P < 0.05, **P < 0.01 (one-way ANOVA followed by Tukey’s multiple comparisons test).

In the later stage of specification of organoid cells, the organoids expanded in 1.5-kPa GelMA-HA-RGD hydrogels were transferred into different stiffness gels with culture medium suitable for differentiation (Fig. 4A) (*15*). After 15 days of culture, the marker genes of HCs (*Myo6*, *Myo7a*, *Slc17a8*, and *Pvalb*) were expressed at the highest levels of organoids in the 5-kPa hydrogel (Fig. 4B). To show the generation of HCs in real time during the differentiation of organoids, we cultured CPCs from *Atoh1*–enhanced green fluorescent protein (EGFP) mice. We observed that the proportion of EGFP^+^ cells in the organoids gradually increased with increasing stiffness of the hydrogel from 0.2 to 5 kPa, which was further confirmed by the proportion of MYO7A^+^ HCs in the organoids. The percentage of EGFP^+^/MYO7A^+^ HCs reached the highest level in the organoids from the 5-kPa hydrogel, which was even higher than in Matrigel (Fig. 4, C to E). Furthermore, CPCs from *Atoh1*CreER-tdTomato mice were seeded in different hydrogels. After 4-hydroxytamoxifen treatment during the initial stage of differentiation, tdTomato^+^ cells could be used to trace the organoid HCs. Nearly all of the tdTomato^+^ cells were labeled by the early HC marker MYO6, while some of the tdTomato^+^ HCs expressed POU4F3 and PARVALMBUMIN (PVALB), which are markers of relatively mature HCs. The proportion of POU4F3^+^ or PVALB + mature HCs in the organoids also increased with increasing stiffness of the hydrogels, reaching the highest proportion in the 5-kPa hydrogel (Fig. 4, F to K). Furthermore, we found that the MYO7A^+^ HCs in organoids from 5-kPa hydrogels displayed HC bundles as indicated by F-actin (Fig. 4L). Furthermore, we observed that cells within the differentiated organoids exhibited positive staining for VGLUT3 (an inner HC marker) and PRESTIN (an outer HC marker) (fig. S2). These results revealed gradient ECM stiffness power sensory epithelium formation in cochlear organoids.

### Cochlear organoids in hydrogels mimic the dynamic formation of the cochlear sensory epithelium

**Fig. 5.**
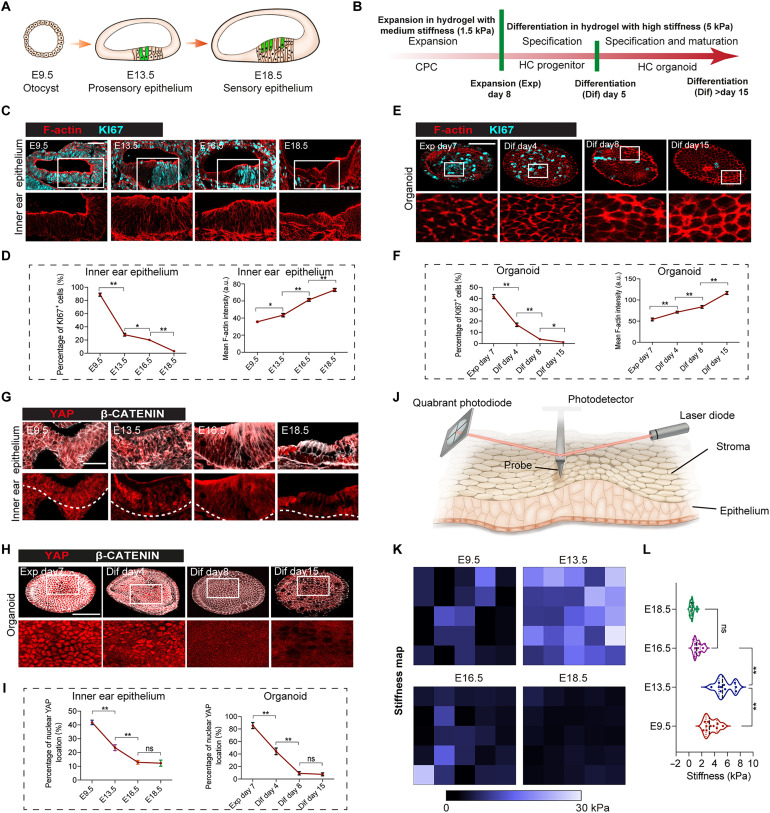
Developing organoids mimic the tissue remodeling of cochlear epithelia during development. (**A**) Schematic depicting the sensory epithelium morphogenesis from E9.5 to E18.5. (**B**) The strategy for generating cochlear organoids from CPCs in a stiffness-tunable hydrogel system. (**C**) Confocal images show F-actin and KI67 staining of the developing inner ear epithelia. Scale bar, 50 μm. (**D**) Three independent experiments have identified F-actin intensity and the percentage of KI67^+^ cells in the indicated area from (C). Six tissues from six embryos at each condition. (**E**) Confocal images show F-actin and KI67 staining of the developing cochlear organoids. Scale bar, 50 μm. (**F**) Three independent experiments have identified F-actin intensity and the percentage of KI67^+^ cells from (E). Six tissues from six embryos at each condition. (**G**) Confocal images show YAP and β-CATENIN staining of the developing inner ear epithelia. Scale bar, 30 μm. (**H**) Confocal images show YAP and β-CATENIN staining of the developing organoids. Scale bar, 50 μm. (**I**) Three independent experiments were done to quantify YAP nuclear localization–positive cells in the indicated area from (G) and (H). Six tissues from six embryos at each condition. (**J**) Schematic depicting the stiffness of the inner ear stromal region close to the epithelial region was detected by AFM. (**K**) Stiffness maps of the stromal regions of the developing inner ear. (**L**) Three independent experiments were done to quantify tissue stiffness. Ten tissues from six embryos at each stage. The data are presented as the means ± SEM, *P < 0.05, **P < 0.01 (one-way ANOVA followed by Tukey’s multiple comparisons test).

To further investigate the roles of the ECM and mechanical force in the sensory epithelium formation during the development of the inner ear in vivo, we correlated the developmental process of the cochlear epithelium (Fig. 5A) with the stages of cochlear organoid formation in the stiffness-shifted hydrogel system (Fig. 5B). The expansion stage of CPCs in the 1.5-kPa hydrogel mimicked the expansion stage of the prosensory region at embryonic day 9.5 (E9.5) to E13.5 and was characterized by the significant increase in proliferating KI67^+^ progenitor cells in the epithelium (Fig. 5, C to F). The specification stage of CPCs in the 5-kPa hydrogel mimicked the specification stage of the sensory region during E13.5 to E18.5 and was characterized by the obvious decrease in KI67^+^ proliferating cells and the gradual increase in MYO7A^+^ HCs (Fig. 5, C to F, and fig. S3A). We detected the dynamic collagen II expression during inner ear development, and a notable cochlear duct was found at E11.5, which is when the presensory epithelium begins to exit the cell cycle (fig. S3B). The expression of collagen II was most prominent on the ends of the epithelium, which suggested that the ECM was remodeled during the critical stages of the CPC fate transition.

It has been reported that actomyosin contractility is the source of cellular mechanical force (*16*) and is the transducer of ECM mechanical force (*17*), and we observed the gradually increased expression of the F-actin along the differentiation process in both the developing inner ear epithelium and organoid (Fig. 5, C to F). As the gel is gradually degraded during organoid differentiation, and the decreasing expression of collagen II along with CPC differentiation was noticed as well, these results indicate a nonlinear relationship between F-actin cytoskeleton enrichment and ECM stiffness, and the sensory epithelium undergoes active F-actin enrichment during differentiation.

We further examined the expression of YAP, a master mechanotransducer in response to ECM mechanical forces, in the process of sensory epithelium morphogenesis. We observed that the nuclear localization of YAP was gradually reduced along with decreased proliferation and enhanced differentiation during sensory epithelium development and organoid formation (Fig. 5, G to I). Furthermore, we observed that the expression of β-CATENIN, the effector of canonical Wnt signaling, also showed the same trend as the active YAP in both the cochlear sensory region and organoids, which might serve as a relayed transducer from the mechanical force to the genetic changes determining the cell fate of CPCs.

On the basis of the above results, we hypothesized that the mechanical cues originating from the stromal region surrounding the epithelium play a crucial role in the development of sensory epithelium. To evaluate this hypothesis, we used atomic force microscopy (AFM) to measure the stiffness of the stromal area connected to the epithelium (Fig. 5J). Using a 2D stiffness map of inner ear tissue sections, we observed that the stroma at E13.5 exhibited higher stiffness compared to other developmental stages, which aligns with the observation that organoid differentiation within the hydrogel system necessitates a higher stiffness of ECM (Fig. 5, K and L). According to the results above, the sensory epithelium development process could be simulated by the cochlear organoid in the current stiffness tunable culture system, both on the ECM remodeling and cellular behavior of progenitor cells.

### Mechanical forces regulate the expansion of prosensory epithelium through ITGA3/F-actin cytoskeleton/YAP signaling

**Fig. 6.**
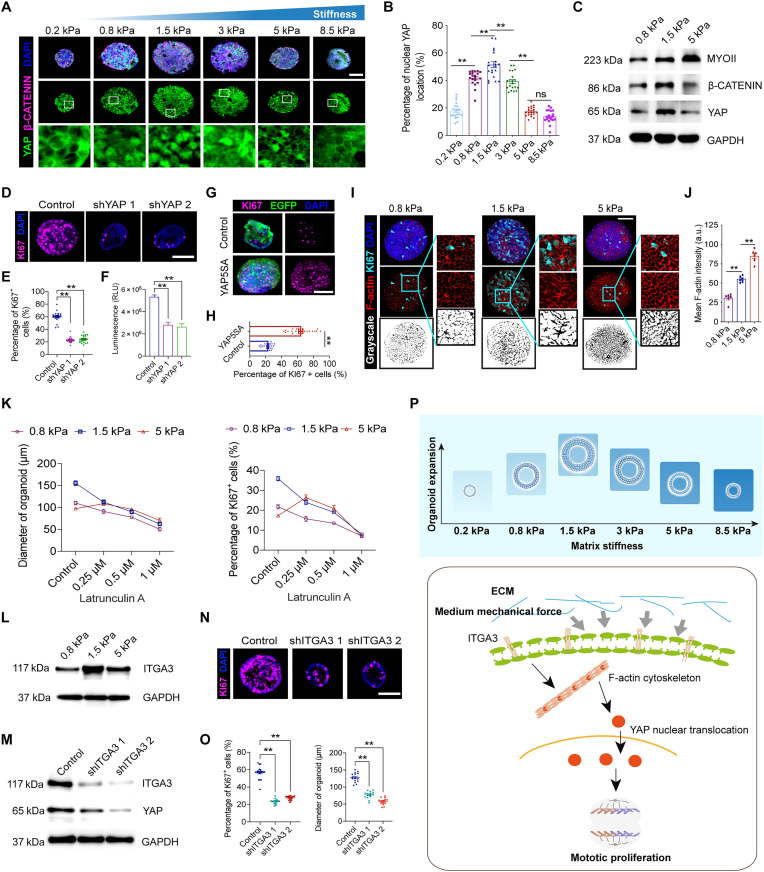
Matrix stiffness requires ITGA3/F-actin cytoskeleton/YAP signaling to promote organoid expansion. (**A**) Confocal images show YAP and β-CATENIN staining. Scale bar, 50 μm. (**B**) Quantifying the percentage of YAP nuclear localization–positive cells per organoid from (A). Twenty organoids at each condition, three independent experiments. (**C**) Western blot analysis showing the indicated proteins. GAPDH was used as the loading control. (**D** and **E**) Confocal images and quantification of KI67 in hydrogels with 1.5 kPa. Scale bar, 50 μm. Nineteen organoids at each condition, three independent experiments. (**F**) Luminescent cell viability assay measuring *YAP* KD CPCs (*n* = 3 independent experiments). (**G** and **H**) Confocal images and quantification of KI67 in 0.8-kPa gels. EGFP indicates lentivirus fluorescence. Scale bar, 50 μm. Twenty-four organoids at each condition, three independent experiments. (**I**) Confocal images showing cytoskeleton F-actin and KI67 staining. Scale bar, 50 μm. (**J**) Three independent experiments identified the intensity of F-actin per organoid from (I). Six organoids at each condition. (**K**) Quantifying the diameter of organoids and the percentage of KI67^+^ cells per organoid in indicated conditions. (**L** and **M**) Western blot analysis showing the indicated proteins in organoids cultured in indicated conditions. GAPDH was used as the loading control. (**N**) Confocal images show KI67 staining in organoids after *ITGA3* KD in hydrogels with 1.5 kPa. Scale bar, 50 μm. (**O**) Quantifying the percentage of KI67^+^ cells per organoid and diameter of organoids (N). Fifteen to 20 organoids at each condition. (**P**) The mechanism behind the ECM medium stiffness modulates the cochlear organoid expansion. The data are presented as the means ± SEM, *P < 0.05, **P < 0.01. One-way ANOVA followed by Tukey’s multiple comparisons test in (B), (E), (F), (J), and (O). Unpaired Student’s *t* test in (H).

To further understand how sensory epithelium generation is controlled by mechanical forces from the ECM, we evaluated the nuclear localization of YAP in the organoids cultured in GelMA-HA-RGD hydrogels of varying stiffness during the stage of CPC expansion. The nuclear localization of YAP did not exhibit a linear increase with increasing matrix stiffness but rather increased from 0.2 to 1.5 kPa and then decreased with higher stiffness (Fig. 6, A to C, and fig. S4A). β-CATENIN also exhibited a similar expression pattern as YAP, which suggests that YAP promotes cell proliferation by up-regulating β-CATENIN as inferred from our previous works (*13*). Genes related to the progenitor cell identity and proliferative capacity of CPCs were measured by real-time polymerase chain reaction (PCR), and most were up-regulated in organoids from the 1.5-kPa hydrogel, which was consistent with the expression of YAP and β-CATENIN as well as with the cell proliferation assay of CPCs (Fig. 6C and fig. S4B). We observed a continuous increase in myosin II (MYO II) expression, which is required for cytoskeleton polymerization, in organoids cultured in hydrogels with increasing stiffness (Fig. 6C). We knocked down (KD) *YAP* using short hairpin RNA (shRNA) in the 1.5-kPa hydrogels to directly test the involvement of YAP signaling in matrix stiffness-induced CPC expansion (fig. S4C), *YAP* KD led to reduced size of the organoids and cell viability, and the number of KI67^+^ CPCs was significantly decreased (Fig. 6, D to F, and fig. S4, D and E). To induce *YAP* overexpression, we transfected CPCs with lentivirus-based *YAP5SA*, a constitutively active version of *YAP*, within 0.8-kPa hydrogels (fig. S4F). The overexpression of *YAP* resulted in increased cell proliferative capacity and cell viability (Fig. 6, G and H, and fig. S4, G to I), providing evidence that *YAP* is an effector of ECM stiffness-mediated cell proliferation.

Similar to the expression pattern of MYO II, the F-actin cytoskeleton showed a stiffness-dependent enrichment as indicated by the level of F-actin intensity (Fig. 6, I and J). To verify that the cytoskeleton enrichment induced by the mechanical force from the ECM regulates the proliferation of CPCs, we used small molecules to interfere with MYO II and F-actin. Blebbistatin, an inhibitor of MYO II, led to a dose-dependent decrease in the size of organoids and the number of KI67^+^ cells in the 0.8- and 1.5-kPa hydrogels, while a low dose (2.5 μM) did not show a notable decrease in the size or number of proliferating cells in organoids cultured in 5-kPa hydrogels (fig. S4, J and L). We next used latrunculin A to directly inhibit cytoskeleton enrichment, and we observed a dose-dependent decrease in organoid size and the number of proliferative cells after treatment with latrunculin A for 48 hours in both the 0.8- and 1.5-kPa hydrogels (Fig. 6K and fig. S4K). A lower concentration of latrunculin A (0.25 μM) induced a prominent elevation in organoid growth in the 5-kPa hydrogel, which might be due to the mild F-actin cytoskeleton depolymerization compensating for the inhibition imposed by the stiff hydrogel.

Integrins play a crucial role in connecting the ECM to the F-actin cytoskeleton, facilitating the transmission of biochemical and mechanical signals between cells and their surrounding environment (*18*). Notably, ITGA3 has been identified as a key regulator of YAP nuclear localization (*19*). In our study, we observed higher expression of ITGA3 in organoids cultured in 1.5-kPa hydrogels compared to those in 0.8- and 5-kPa hydrogels (Fig. 6L, fig. S4, M and N). To investigate whether ITGA3 mediates the link between medium ECM stiffness signals and YAP activation, thereby promoting cell proliferation, we used *ITGA3* KD in CPCs within the 1.5-kPa hydrogels (Fig. 6M). ITGA3 deficiency resulted in a decrease in cell proliferation capacity and reduced cell activity (Fig. 6, N and O, and fig. S4, O and P), accompanied by a decrease in YAP expression (Fig. 6M). Together, these results suggest that ITGA3/F-actin cytoskeleton/YAP cascades serve as the transducers that convert the mechanical forces from the ECM into genetic cascades that control the expansion stage of the prosensory epithelium (Fig. 6P).

### Increased mechanical forces from the ECM promote the sensory epithelium formation through Ca^2+^/ERK1/2/KLF2 signaling

**Fig. 7.**
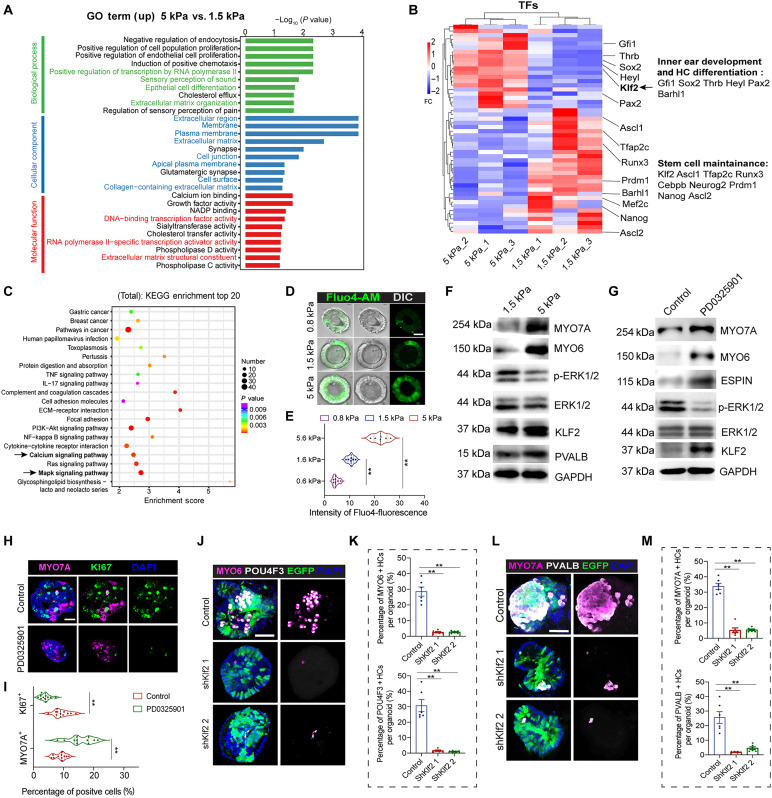
Matrix stiffness drives organoid cells specialization into HCs by modulating ERK signaling–mediated Klf2 activation. (**A**) The top 30 GO terms of the up-regulated genes related to organoids in 5-kPa gels versus 1.5-kPa gels. (**B**) Heatmap showing the differentially expressed TFs between organoids in 5-kPa gels and 1.5-kPa gels. (**C**) KEGG pathway enrichment shows the DEGs between organoids in 5-kPa gels and 1.5-kPa gels. (**D**) Fluorescence and differential interference contrast (DIC) images show the Fluo4-AM intensity of organoids cultured in three different stiffness gels. (**E**) Quantification of the Fluo4-AM intensity of the organoids. Six organoids at each condition, three independent experiments. (**F**) Western blot analysis of the indicated proteins in organoids from two different stiffness hydrogels. GAPDH was used as the loading control. (**G**) Western blot analysis of the indicated proteins in PD-treated and control organoids in medium-stiffness gels. GAPDH was used as the loading control. (**H** and **I**) Confocal images and quantification of MYO7A and KI67 staining in PD-treated and control organoids. Scale bar, 50 μm. Eleven organoids at each condition, three independent experiments. (**J** and **K**) Confocal images and quantification of MYO6 and POU4F3 staining of organoids after *Klf2* KD in stiff gels. EGFP indicates lentivirus fluorescence. Scale bar, 50 μm. Six organoids at each condition, three independent experiments. (**L** and **M**) Confocal images and quantification of MYO7A and PVALB in organoids after *Klf2* KD in stiff gels. EGFP indicates lentivirus fluorescence. Scale bar, 50 μm. Six organoids at each condition, three independent experiments. The data are presented as the means ± SEM, *P < 0.05, **P < 0.01. One-way ANOVA followed by Tukey’s multiple comparisons test in (E), (K), and (M). Unpaired Student’s *t* test in (I).

In the differentiation medium, organoids were cultured for 5 days in 1.5- and 5-kPa hydrogels. RNA sequencing was performed to analyze the differences in transcriptome signatures between organoids in the two hydrogels. The principal components analysis plot indicated that the replicates of each group were well clustered (fig. S5A). The number of differentially expressed genes (DEGs) with their fold change (FC) is shown in fig. S5B. We identified 255 up-regulated DEGs and 663 down-regulated DEGs in organoids cultured in 5-kPa hydrogels versus 1.5-kPa hydrogels, and Gene Ontology (GO) analysis of DEGs with FC >1.5 showed enrichment of genes related to the sensory perception of sound, epithelial cell differentiation, and ECM organization (Fig. 7A and fig. S5C). In addition, DNA-binding transcription factor (TF) activity was also found among the up-regulated GO terms. Because TFs are the crucial determinants for regulating gene expression programs that determine cell fate, we analyzed the differentially expressed TFs between those two groups. We found that *Gfi1*, *Sox2*, and *Pax2*, which are involved in inner ear development and HC differentiation, were up-regulated in the 5 kPa group (Fig. 7B). TFs associated with stemness, such as *Klf2*, *Ascl1*, *Nanog*, and *Ascl2*, were also differentially expressed, and *Klf2* was up-regulated by eightfold in the organoids cultured in 5-kPa hydrogels during differentiation. The KEGG pathway analysis showed DEGs (FC >1.5) enriched in the mitogen-activated protein kinase (MAPK) pathway, Ras pathway, and focal adhesion pathway (Fig. 7C). In addition, DEGs enriched in the Calcium signaling KEGG pathway were up-regulated in the organoids cultured in 5-kPa hydrogels, while ECM-receptor interaction was down-regulated (fig. S6, A and B).

Given that the MAPK/ERK pathway acts as a transducer responsible for converting mechanical force from the ECM into intracellular signaling via Ca^2+^ signaling (*20*), and because *Klf2* can also transduce the mechanical forces, particularly the shearing stress, we hypothesized that MAPK/ERK signaling and *Klf2* are crucial effectors for promoting HC differentiation in hydrogels with greater mechanical forces. The calcium signal was notably increased with the increasing stiffness of the hydrogel during organoid specialization, which was further verified by Fluo4-AM staining and real-time PCR assay (Fig. 7, D and E, and fig. S6C). Western blot confirmed that cells in organoids in the 5-kPa gel tended to differentiate into HCs as indicated by the elevated expression of the HC markers MYO7A, MYO6, and PVALB (Fig. 7F). The suppressed ERK1/2 and activated KLF2 expression in the stiffer gels further supported the hypothesis that the MAPK/ERK pathway and KLF2 direct the differentiation of organoids.

To precisely evaluate the roles and relationship of the MAPK/ERK pathway and KLF2 in HC differentiation, PD0325901 (PD), an inhibitor of ERK1/2, was applied during organoid differentiation for 10 days in 1.5-kPa hydrogels (Fig. 7G). We observed significantly more MYO7A^+^ HCs and fewer KI67^+^ proliferating cells within the organoids after treatment with PD (Fig. 7, H and I). Western blot further confirmed the up-regulation of HC markers along with the down-regulation of ERK1/2 after treatment with PD (Fig. 7G). The expression of KLF2 was also up-regulated by PD, which suggested that KLF2 serves as a downstream effector of the MAPK/ERK pathway. To further investigate the role of *Klf2* in sensory HC generation, we KD *Klf2* by shRNA (fig. S7A), and we observed a notably reduced number of HCs in organoids cultured in 1.5-kPa hydrogels (Fig. 7, J to M). In addition, the promoted HC differentiation by PD was blocked by *Klf2* KD (fig. S7B). These results show that greater mechanical forces from the ECM promote the differentiation of HCs through Ca^2+^/ERK1/2/KLF2 signaling.

### PIEZO2 senses the ECM mechanical forces to drive sensory epithelium formation

**Fig. 8.**
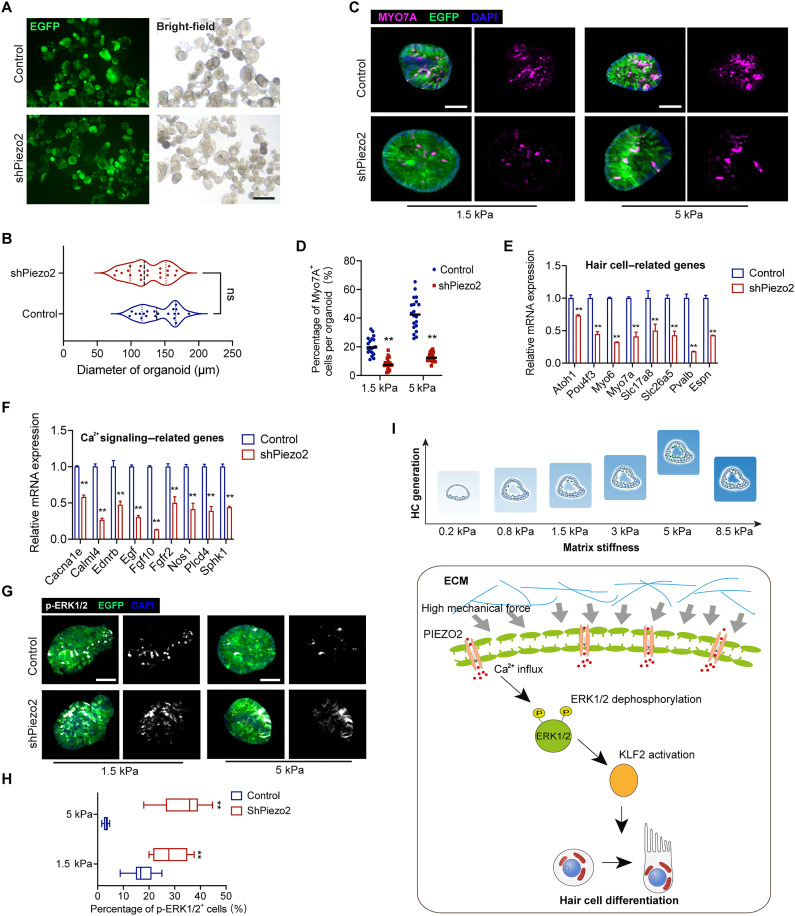
PIEZO2 senses matrix stiffness to drive organoid specialization. (**A**) Fluorescence and DIC show organoid diameter after *Piezo2* KD in 5-kPa gels. EGFP indicates lentivirus fluorescence. Scale bar, 250 μm. (**B**) Quantifying the diameter of organoids from (A). Twenty organoids at each condition, three independent experiments. (**C** and **D**) Confocal images and quantification of MYO7A staining of organoids after *Piezo2* KD in 1.5- and 5-kPa gels. EGFP indicates lentivirus fluorescence. Scale bar, 50 μm. Twenty organoids at each condition, three independent experiments. (**E** and **F**) Real-time PCR analysis shows the relative expression of HC-related and Ca^2+^-related genes after *Piezo2* KD in 5-kPa gels. The results were normalized to *Gapdh* in the same sample and then normalized to the control group (*n* = 3). (**G**) Confocal images show p-ERK1/2 staining of organoids cultured in 1.5- and 5-kPa hydrogels. EGFP indicates lentivirus fluorescence. Scale bar, 50 μm. (**H**) Quantifying percentage of p-ERK1/2–positive cells per organoid from (G). Ten organoids at each condition, three independent experiments. The data are presented as the means ± SEM, *P < 0.05, **P < 0.01, *t* test. (**I**) The mechanism behind the higher stiffness from ECM modulates the cochlear organoid specialization. The data are presented as the means ± SEM, *P < 0.05, **P < 0.01 (unpaired Student’s *t* test).

How progenitor cells sense higher stiffness to differentiate into sensory cells remains to be explored. It has been reported that PIEZO1/2 is required to activate intracellular Ca^2+^ in response to mechanical stimulation (*21*). We found that the expression *Piezo2*, but not *Piezo1*, was up-regulated in the stiffer gels (fig. S6D). We hypothesized that PIEZO2 served as a mechanosensor for detecting high stiffness and converting mechanical forces into intracellular Ca^2+^ signals. To test this hypothesis, we designed lentivirus-based shRNA to KD *Piezo2* in individual CPCs (fig. S8A), and no noticeable difference in cell expansion was found after 8 days in the 1.5-kPa hydrogel (Fig. 8, A and B). Subsequently, organoids were transferred into the 1.5-kPa hydrogel and 5-kPa hydrogel for further differentiation. After 15 days, we found that KD *Piezo2* offsets the effect of MYO7A^+^ HC generation induced by increased stiffness (fig. S8B, Fig. 8, C and D). The expression of HC-related genes was also significantly decreased by inhibited *Piezo2* in the 5-kPa hydrogel (Fig. 8E).

We further detected whether Piezo2 mediates Ca^2+^ signaling in the context of stiffness-triggered HC generation. As shown in Fig. 8F, Ca^2+^ signaling–related genes were significantly decreased by *Piezo2* KD. The matrix stress–suppressed phosphorylation of ERK1/2 was reversed in the 1.5- and 5-kPa hydrogel (Fig. 8, G and H). In addition, KLF2 protein expression was reduced by *Piezo2* KD (fig. S8A). Together, the higher stiffness from ECM was sensed by PIEZO2 and then triggered the intracellular Ca^2+^/ERK1/2/KLF2 cascades to facilitate sensory HC generation (Fig. 8I).

## DISCUSSION

According to the results, the chemically defined GelMA-HA-RGD hydrogel has excellent biocompatibility. Furthermore, its stiffness could be finely tuned to facilitate cochlear organoid formation. This hydrogel system allows us to study how mechanical force regulates sensory epithelium formation. Apart from its excellent solubility and low immunogenicity, GelMA was selected as the essential component due to its outstanding mechanical manipulation properties (*22*, *23*). Although there is an inherent RGD tri-amino acid sequence in GelMA for binding to cell adhesion molecules (*24*–*26*), this does not provide adequate cellular adhesion to enhance the viability of cultured CPCs. Thus, we supplied extra RGD to improve the biocompatibility of the hybrid hydrogel. Soluble HA forms viscoelastic hydrogels when swelling with water and exhibits a high tendency to generate compressive osmotic forces (*27*). A recent study revealed essential roles for extracellular HA in the morphogenesis of the inner ear (*28*). Thus, we supplied HA to provide a more stable mechanical force during the long-term culture after seeding CPCs. HA imparts physical stability to hydrogels, which might be attributed to the interactions between HA and GelMA, reinforcing the 3D networks of the hydrogel scaffolds. Furthermore, the water retention capacity of HA slows down the infiltration of enzymes secreted by cultured cells into the hydrogel network, resulting in slower degradation of the hydrogels.

Compared to the Matrigel-based culture of cochlear organoids (*15*, *29*), manipulating the mechanical parameters in our hybrid hydrogel allows CPCs to proliferate and form cochlear organoids with comparable efficiency to those cultured in Matrigel but with higher HC differentiative efficiency than in Matrigel. In addition, our hybrid hydrogel allows us to closely mimic the dynamic mechanical force of the ECM to determine cell fate during sensory epithelium development. These findings suggest that the hybrid hydrogel system has more practical value and can be used as an alternative matrix to Matrigel for culturing cochlear organoids. Such a hybrid hydrogel with high biocompatibility and tunability of mechanical parameters opens the possibility for wide application with various tissue-derived organoids. Studies are needed to compare the advantages with other hydrogels, such as synthetic poly(ethylene glycol) hydrogels (*30*, *31*), for culturing various organoids.

Our results show that F-actin is the bridge for transducing the intracellular signals for cell expansion within a specific range of ECM stiffness. The driving force of sensory epithelium formation is attributed to the ECM mechanical cues, and most studies suggest that tissue morphogenesis is driven by cellular deformations induced by active contractile actomyosin networks (*16*, *32*). We found that ECM stiffness directly influences cell expansion and specification in the cochlear organoid system and that ECM stiffness promotes F-actin enrichment from 0.8 to 5 kPa in the expansion medium. During the sensory HC generation stage, continuously enhancing F-actin cytoskeleton enrichment was accompanied by decreasing matrix stiffness. This may seem contradictory, but our results prove that passive contractile actomyosin is the mechanism through which ECM stiffness regulates cell expansion. Enhanced F-actin expression might play a role in cell shaping after cell fate determination, such as planar cell polarity organization (*33*) and hair bundle shaping (*34*). Mechanotransduction signaling can convert extracellular mechanical forces into intracellular cell–specific transcriptional programs. A series of studies found that YAP-dependent mechanotransduction signaling mediates a wide range of mechanical stresses from the matrix, including stiffness (*35*, *36*). Different from the theory that increasing matrix stiffness persistently modulates cell behavior through YAP (*37*, *38*), we show here that matrix stiffness regulated CPC proliferation through YAP in the 3D hybrid gels, and higher stiffness resulted in decreased YAP nuclear localization. A recent study also clarified that YAP nuclear localization positively correlates with matrix stiffness in 2D culture. In contrast, the relationship between stiffness and the nuclear localization of YAP is much more complex in 3D culture (*39*). The results of these different experiments reflect the complicated influence and mechanisms by which different degrees of matrix stiffness regulate cell behavior. Researchers should ensure that they choose a reasonable culture method when matrix stiffness is involved.

We further showed that stabilized KLF2 and inhibited ERK1/2 phosphorylation could relay the mechanical force to promote CPC differentiation into HCs. Unlike 2D stiff substrates enhance ERK activity in adherent pluripotent stem cells (*40*, *41*), mechanical stress from the 3D hydrogels triggered suppressed stiffness-sensitive ERK signaling in CPCs. To possibly explain this discrepancy, we propose that the stiffness of the 2D matrix sets a relatively wide span for these studies, and the reactivity of cells in different ranges of mechanical stress will be different. How cells sense pro-differentiation mechanical forces to provoke intracellular calcium signaling remains to be resolved. The PIEZO mechanosensitive channel is required for the mechanical activation of cytosolic Ca^2+^ to drive stem cell differentiation in the adult midgut (*21*), and PIEZO is a central modulator of mechanical stress during heart valve development by controlling KLF2 activity to guide endothelium morphogenesis (*42*). Our results suggest that PIEZO2 might act as a critical sensor of mechanical signaling during HC generation, and this needs to be further verified through genetic interventions in vivo. KLF2 is a mechanosensor of mechanical forces that exclusively senses the shear stress exerted by blood flow in the cardiovascular system (*43*, *44*), and our results suggest that KLF2 is an effector of ECM stiffness that promotes CPC differentiation into HCs. Further studies should explore the potential and connection between calcium signaling and KLF2 activity in inner ear development and HC regeneration in vivo.

In the current study, we created a stiffness-tunable hydrogel system for cochlear organoid culture. We showed how the ECM mechanical forces were converted into cellular signals and direct sensory epithelium in vitro. This study opens avenues for defining alternative culture systems to better manipulate stem/progenitor cell fate through modulation of the mechanical properties of the system. The hybrid hydrogels described here can be adapted to match the mechanical characteristics of the niches in which stem/progenitor cells reside. Moreover, the mechanism through which mechanical forces regulate cell fate provides potential targets for regenerative medicine.

**Fig. 9.**
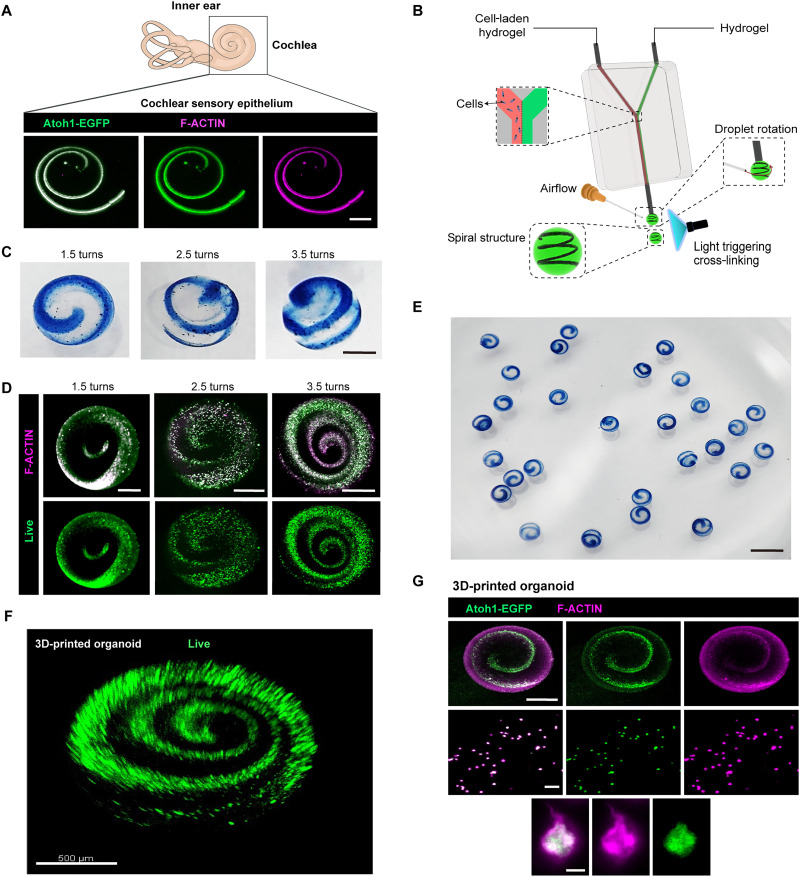
3D-printed spiral organoids mimic the structure of the cochlear epithelium. (**A**) Confocal images showing the structure of cochlear epithelium by F-actin staining and Atoh1-EGFP. Scale bar, 300 μm. (**B**) Schematic of the bioprinting device components and process. (**C**) 3D-printed microspheres with different turns. Blue ink shows the printed CPCs. Scale bar, 300 μm. (**D**) 3D-printed hydrogel microspheres with different numbers of turns of CPCs. Scale bar, 300 μm. (**E**) 3D-printed microspheres with 2.5 turns. Blue ink marks the CPCs. Scale bar, 1200 μm. (**F**) 3D reconstruction of live staining of the 3D-printed cochlear organoids at culture day 7. Scale bar, 500 μm. (**G**) Confocal images of Atoh1-EGFP and F-actin staining of 3D reconstruction of the 3D-printed cochlear organoids on culture day 7. Scale bars, 300 μm (top), 50 μm (middle), and 4 μm (bottom).

The cochlear organoids cultured in the hybrid hydrogel system were morphologically similar to those grown in Matrigel. Current methods produce self-organized organoids that are closed cyst-like structures that differ significantly from the spiral-shaped sensory epithelium of the cochlea in vivo (Fig. 9A). Here, we developed a 3D bioprinting technology for spiral microspheres with stable circles using GelMA-HA-RGD gels as the bio-ink scaffolds (Fig. 9, B to E). We envision that such gels more precisely model the environmental and structural factors needed for the culture of other organoids with excellent rheological properties (such as shear thinning), thermo-sensitive characteristics (fig. S1, B and C), and rapid prototyping properties. While the spiral cochlear organoids demonstrate high viability and contain HC-like cells (Fig. 9, F and G), they currently lack tightly connected epithelia and microenvironments that facilitate diverse cellular interactions. Therefore, further efforts should be directed toward enhancing the mimicry of physiological features and achieving functionalization, such as by constructing tissue-level networks for cell-cell communication, which will enable the vascularization and neuralization of organoids.

## MATERIALS AND METHODS

### Animals

*Lgr5*-EGFP-IRES-creERT2, *Atoh1*-EGFP, and *Atoh1*-CreER; Rosa26-tdTomato mice were used in the study. The sources of animals are listed in table S1. Genotyping of transgenic mice is described in table S2. The animals were bred in the Fudan University Animal Facility to acquire pups and embryos. The feeding room was maintained at a controlled temperature of 22 ± 1°C, a humidity of 30 to 70%, and a 12-hour light-dark cycle. Animal Research: Reporting of In Vivo Experiments guidelines were followed in the present study. All animal experiments were approved by the Institutional Animal Care and Use Committee of Fudan University.

### Preparation and synthesis of the hybrid hydrogel

A total of 5 g of gelatin (type A, 300 bloom, Sigma-Aldrich, St. Louis, MO, USA) was dissolved in 45 ml of PBS at 60°C under stirring to obtain a 10% w/v gelatin aqueous solution. Different volumes of methacrylic anhydride (denoted as MA, Sigma-Aldrich, St. Louis, MO, USA) were added to the gelatin solution at 0.5 ml/min under stirring at 50°C to prepare GelMA with different ratios of MA. After reaction in the dark for 3 hours, the products were diluted fivefold with warm PBS (50°C) and dialyzed against PBS for 7 days at 40°C using a dialysis membrane to remove salts and excess free MA. The products were then lyophilized for 2 days to obtain white porous foam and stored at −20°C until further use.

GelMA, HA, RGD, and the photoinitiator lithium phenyl-2,4,6-trimethyl-benzoylphosphinate were mixed in PBS at 37°C, and the prepolymer solution was sterilized by filtering through a 0.22-μm filter before use. Cells were added to the prepolymer solution and placed in molds for culturing, and a 405-nm wavelength light was used to cross-link the prepolymer solution.

### H nuclear magnetic resonance

H NMR spectra were analyzed using an NMR spectrometer (JEOL) with a single-axis gradient inverse probe at a frequency of 300 MHz. Before the measurement, 20 mg of GelMA macromers were completely dissolved in 1 ml of deuterium oxide containing 0.05% (w/v) 3-(trimethylsilyl) propionic-2,2,3,3-d4 acid sodium salt for calibration (Sigma-Aldrich). The gelatin without functionalization was also examined for calculating the degree of substitution (DoF) of MA using the following equation:

DoF=1−(lysine methylene proton of GelMA)/(lysine methylene proton of Gelatin)×100%

### 2,4,6-Trinitrobenzene–sulfonic acid assay

The assay was performed to determine GelMA’s degree of substitution. Briefly, GelMA and gelatin samples were first dissolved in 0.1 M sodium bicarbonate buffer at 1.6 mg/ml and then mixed with an equal volume (0.5 ml) 0.01% 2,4,6-trinitrobenzene–sulfonic acid solution and incubated for 2 hours. The reaction was stopped by adding 0.25 ml of 1 M HCl and 0.5 ml of 10% (w/v) SDS. Each sample’s amino group concentration (AC) was determined from a glycine standard curve after measuring their absorbance at 335 nm. The degree of saturation (DS) (61.2%) was calculated according to the current formula:

DS(%)=(1−AC of GelMA/AC of gelatin)×100%

### Scanning electron microscopy

To examine the micromorphology of the hydrogel array, the gels were dried in a vacuum-drying machine at −80°C for 24 hours. The samples were then treated with a metal spraying machine and observed under a scanning electron microscope (Zeiss Crossbeam 340, Carl Zeiss AG, Oberkochen) at an operating voltage of 2 kV. ImageJ analyzed the pore size.

### Mechanical property evaluation

The storage modulus of the cross-linked hydrogels with different GelMA concentrations was measured for subsequent cell culturing. The hydrogels were characterized by rheology using an Anton Paar MCR302 rheometer with a parallel plate, and a solvent trap was used to ensure temperature stability. A volume of 0.3 ml of hydrogel was incubated at 37°C for 30 min on the rheometer surface and then using a 405-nm band light curing for measurement. The time-varying examination was conducted with an angular frequency of 314 rad/s and 1% strain. Similarly, a volume of 0.3 ml of Matrigel was incubated at 37°C for 30 min on the rheometer surface for solidification and then measured storage modulus under the same parameters.

Cells were encapsulated in the cross-linked hydrogels for the degradation assay and cultured for 0, 1, 2, 3, 4, and 5 days. Hydrogel cylinders were fabricated in a ∅9 mm by 6.3 mm mold, and the cross-linked hydrogels were compressed under a stress-strain curve testing machine (UTM-2203, Shenzhen Suns Technology) with a load capacity of 20 N.

The viscosity and temperature-varying storage modulus were measured in uncross-linked hydrogels. A volume of 0.3 ml of hydrogel was incubated at 37°C for 30 min on the rheometer surface. The viscosity of hydrogel was performed between shear rates of 1 and 100 s^−1^ at 37°C. For temperature sweep, a volume of 0.3 ml of hydrogels was performed between the temperature of 10° and 37°C with an angular frequency of 314 rad/s and 1% strain.

### Release quantification analysis

A hydrogel prepolymer containing rhodamine (40 mg/ml) was used to fabricate microspheres with diameters of about 1260 μm. The microspheres were centrifuged at 1000 rpm for 5 min at room temperature and then immersed in 1 ml of DPBS. The release quantity was examined by fluorescence microscopy at 0, 20, 60, 120, and 240 min after immersing in DPBS. The tested DPBS was compensated to the original volume after every test. For qualitative image analysis (all confocal figures shown in the manuscript), confocal images recorded under optimum confocal settings were presented as selected by the confocal system (pinhole and gain setting). ImageJ was used to measure each microsphere’s total fluorescence intensity for rhodamine.

### Water absorption capability

The water absorption property of the gels was determined by the mass method. Gels with the same volume were cured and placed in an oven for drying at 60°C for 24 hours. The dried gels were placed in deionized water at 20°C, taken out at different intervals, and put on a stainless steel net for 30 s to remove the surface water and weighed.

The following formula was used to calculate the change in sample water absorption over time:

$$Q_{t}=(Wt-W2)/W2$$

*W*2 refers to the dry mass of the original sample, and *Wt* refers to the mass of the sample soaked in water for 24 hours.

### Culture of CPCs from the neonatal cochlea

The cochleae of mice were dissected at postnatal day 0 (P0) to P1 and collected in ice-cold PBS (Thermo Fisher Scientific). The sensory epithelia were detached from the stria vascularis and modiolus by sterile forceps and then incubated in 0.125% trypsin-EDTA for 15 min at 37°C (Thermo Fisher Scientific). Digestion was terminated by trypsin neutralizer (Thermo Fisher Scientific) followed by gentle trituration with a 1-ml syringe. The cell suspension was then passed through a 40-μm cell strainer, and the cells were washed with PBS two times. After centrifuging at 1500 rpm for 6 min, the cells were collected after removing the supernatant. To isolate Lgr5-positive cells, the collected cells derived from *Lgr5*-EGFP-IRES-creERT2 mice were stained with 4′,6-diamidino-2-phenylindole (DAPI). Then, the stained cells were sorted using a BD Influx cell sorter (BD Biosciences) through fluorescence-activated cell sorting to obtain single, live EGFP^+^ cells. For Matrigel culturing, cells were resuspended in 50% Matrigel (Corning), and a droplet of 50 μl of Matrigel (Corning) was placed at the bottom of a 24-well plate. After solidifying in a 37°C incubator for 30 min, the culture medium was added. For hybrid hydrogel culturing, the cells were resuspended in GelMA hydrogels with different ratios of the components (varying amounts of GelMA, HA, and RGD). The 200-μl-cell–laden hydrogels were planted on solidifying rings. The rings were placed in a 24-well plate after rapid cross-linking under light irradiation, and the culture medium was added to the plates. The expansion medium was based on Dulbecco’s modified Eagle’s medium/nutrient mixture F-12 (Thermo Fisher Scientific) supplemented with 1% N2 (GIBCO), 2% B27 (GIBCO), and penicillin-streptomycin (100 μg/ml), epidermal growth factor (50 ng/ml, Proteintech), basic fibroblast growth factor (50 ng/ml, Proteintech), insulin-like growth factor (50 ng/ml Proteintech), 3 μM CHIR99021 (Sigma-Aldrich), 500 μM valproic acid (Sigma-Aldrich), 2-phospho-l-ascorbic acid (100 mg/ml, Sigma-Aldrich), and 2 μM 616452 (Sigma-Aldrich) (*15*). After 8 to 10 days of expansion, the combination of growth factors and small molecules was changed into a differentiation medium containing 3 μM CHIR99021 (Sigma-Aldrich) and 5 μM LY411575 (Selleck). As indicated, the following small molecules were supplied to the culture media at the specified concentrations: blebbistatin (2.5, 5, and 10 μM, Sigma-Aldrich), latrunculin A (0.25, 5, and 10 μM, Sigma-Aldrich), and PD0325901 (2 μM, Sigma-Aldrich). For the cell proliferation assay, EdU was supplied at a concentration of 5 μM for 4 hours of prefixation.

### Bioprinting of cochlear spiral microspheres

We designed a printing system for manufacturing multiscale heterogeneous spiral microspheres by creating a potential difference between the microsphere printing nozzle and the receiver plate, the formation of microspheres using electrostatic field force, and the flow-assisted device to achieve the dynamic rotation-forming process of microspheres. A pneumatic device for active rotation of the microspheres was added at the end of the microsphere printing nozzle to allow the controllable molding of 3D structures such as spirals and helices of cells in the microspheres.

Cell-laden hydrogels and empty hydrogels were pumped using syringe pumps into metal capillaries that were inserted at the inlet orifices of a microfluidic chip. The microfluidic chip had “Y”-shaped microchannels that allowed two parallel bio-inks to be extruded from a single nozzle, and the microfluidic nozzle could maintain a laminar flow. The material with less proportion of hydrogel could be stretched into a spiral during spinning by adjusting the airflow from a gas-flow needle. The spiral’s geometric parameters, including the number of turns, could be adjusted by changing the injection ratio of the hydrogel in the microchannels and air pressure. After generating the spiral architecture, the patterned droplet could be cross-linked by 405 nm of light. Thus, the liquid droplets could mimic the morphology of the inner ear structure.

We fabricated spiral microspheres with different numbers of spiral turns at 26°C. The diameter of the gas flow needle was 0.3 mm, and the flow velocity of the hydrogel was 0.8 ml/min. The numbers of helical turns in the microspheres were 1.5, 2.5, and 3.5, and the corresponding pressures of the gas flow needle were 56.4, 66.1, and 70.2 kPa. The corresponding flow velocities of cell-laden GelMA were 0.06, 0.08, and 0.1 ml/min, respectively.

### Cell viability assay

Cell viability was assessed by the Live/Dead staining kit (Thermo Fisher Scientific) and CellTiter-Glo 3D cell viability assay (Promega). The CPCs cultured in Matrigel were released using Cell Recovery Solution (Corning), and cells cultured in the hybrid hydrogel were released using GelMA lysis solution. The cells in the organoids were then incubated with 1 μl of Live/Dead reagent (an indicator of dead cells) in the expansion medium for 30 min and then imaged under a Leica DMi8 microscope with 488/570-nm excitation wavelength. The number of dead cells was counted per organoid using ImageJ (National Institutes of Health, USA). For the CellTiter-Glo 3D cell viability assay, the collected cells were incubated with the working fluid for 10 min, and then the cell suspensions were transferred into a black light-proof 96-well plate. Cell viability was recorded by a Tecan M1000 reader according to the manufacturer’s protocol.

### Lentivirus production and transduction

The single-stranded DNA oligonucleotide was annealed to double-stranded DNA. The double-stranded shRNA oligo was inserted into the pSLenti-U6-shRNA–cytomegalovirus (CMV) vector to construct the shRNA recombinant plasmid that was used to transform competent DH5 cells. Plasmid-linked puromycin (Puro) was used to screen cells labeled with Puro, and EGFP was linked to indicate the transfected cells. For shRNA recognizing *YAP*, *Klf2*, and *Piezo2*, the two sequences validated for KD and the one nontarget control shRNA sequence are shown in table S3. For *YAP5SA* transfected experiments, CPCs were transfected with empty lentivirus vector (pcSLenti-EF1-EGFP-P2A-Puro-CMV-MCS-3xFLAG-WPRE) or *YAP5SA* lentivirus. Cochlear organoids from the expansion stage were mechanically broken into small fragments. Lentiviral particles were added to the cell suspension, and polybrene was added at 5 μg/ml. The mixture was centrifuged for 90 min at 600 rpm. The supernatant was removed carefully with a pipette, and the cells were resuspended in hybrid hydrogel and immersed in an expansion medium. mCherry and eGFP expression in CPCs were visualized 48 hours after the transduction.

### Intracellular Ca^2+^ imaging

The intracellular Ca^2+^ concentration was measured using the Ca^2+^-sensitive fluorescent dye Fluo4-AM (Dojindo). The organoids cultured in hybrid hydrogel were washed with Ca^2+^-free Hanks’ balanced salt solution three times before being loaded with 5 μmol Fluo4-AM solution for 30 min at 37°C in the dark. The fluorescence was excited at 480 nm, and emission was detected between 510 and 550 nm. The images were captured with an Sp8 confocal microscope (Leica), and fluorescence intensity was analyzed in ImageJ.

### Immunofluorescence staining

CPC-derived organoids cultured in Matrigel were fixed in 4% paraformaldehyde (PFA) for 1 hour on ice. Hybrid hydrogel-cultured organoids were released by lysate solution and then fixed in 4% PFA for 1 hour. After washing with PBS three times, the organoids were incubated in PBS with 1% Triton X-100 and 5% bovine serum albumin for 2 hours at room temperature for blocking and permeabilization. After a brief wash with PBS, the organoids were transferred to adhesive slides. For cochlear tissue staining, the cochlear tissues at the indicated embryonic stages were dissected and fixed in 4% PFA. After dehydration with a gradient of sugar solutions (10, 20, and 30%), the tissues were encapsulated in an optimal cutting temperature compound (OCT), and frozen sections were prepared. The tissue slides were dried on a drying machine for 30 min, and the samples were subsequently blocked and permeabilized as organoids. The samples were incubated with primary antibodies diluted in PBS with 1% Triton X-100 overnight at 4°C. The primary antibodies are shown in table S4.

After washing with PBS three times, the samples were incubated for 6 hours at room temperature with Alexa 488–, 555–, or 647–conjugated anti-rabbit or anti-mouse secondary antibodies (Invitrogen) at 1:400 dilution in PBS with 1% Triton X-100 overnight. ActinRed 555 Probe Reagent was used to stain the cytoskeleton F-actin, and the nuclei were stained with DAPI (Sigma-Aldrich). The EdU staining was performed using the Click-iT cell proliferation kit (Invitrogen) according to the manufacturer’s protocol. Images were acquired on an Sp8 confocal microscope (Leica) and analyzed in ImageJ. The fluorescence intensity and the positively stained cells were calculated manually with ImageJ. The percentage of positive cells was defined as the ratio of positive cells to the total cells in each organoid. The cell-located region was circled and then the area and fluorescence intensity were calculated by ImageJ. The mean fluorescence intensity was obtained by the ratio of the fluorescence intensity to the area.

### Protein extraction and Western blot

The total proteins in the collected organoids were extracted with the Total Protein Extraction Kit (Beyotime Biotechnology). After incubation in lysis solution for 30 min, the samples were centrifuged at 4000 rpm to acquire the supernatants. After the protein concentrations were determined by the bicinchoninic acid protein assay kit (Thermo Fisher Scientific), the supernatants were mixed with 20% loading buffer (Beyotime Biotechnology) and incubated at 95°C for 10 min. The samples were loaded onto SDS–polyacrylamide gel electrophoresis gels (Beyotime Biotechnology) for electrophoresis and then the proteins in the gels were transferred to polyvinylidene difluoride membranes (Merck Millipore). For blocking, the membranes were incubated with 5% nonfat dry milk for 1 hour at room temperature and then incubated with primary antibodies at 4°C overnight. The primary antibodies used are shown in table S4.

After washing with PBST (0.05% Tween-20 in PBS) for 10 min three times, the membranes were incubated with the appropriate secondary antibody for 2 hours at room temperature (Beyotime Biotechnology). The membranes were washed three times with PBST, incubated with an enhanced chemiluminescence Western blotting kit (Thermo Fisher Scientific), and scanned using an Azure c300 Chemiluminescent Western Blot Imaging System.

### Transmission electron microscopy

Organoids at day 8 of the proliferative stage were released from the 1.5-kPa hydrogel and fixed in 2.5% glutaraldehyde and 2% PFA diluted in 0.1 M phosphate buffer for 8 hours. After washing in PB and dehydration in an ethanol gradient, the samples were embedded in epoxy resin (Sigma-Aldrich). Samples were sectioned at a thickness of 50 nm and mounted on copper grids. After staining with 4% uranyl acetate for 5 min, the sections were stained with 0.4% lead citrate for 1 min. The ultrastructure of the sections was visualized with a transmission electron microscope (CM120, Philips).

### AFM measurements

Fresh inner ear tissues from mice (E9.5, E13.5, E16.5, and E18.5) were embedded in OCT without fixation. Then, the samples were snap-frozen and sectioned into 40-μm-thick sections with a microtome. Sections were placed onto poly-lysine–coated slides and covered with PBS. Before AFM measurements, each sample was stained with Hoechst 33342 to label cell nuclei. Samples were mounted on an atomic force microscope system (JPK NanoWizard ULTRA Speed 2; Bruker) and subjected to tissue stiffness measurements. Silicon nitride cantilevers with a spring constant of 0.06 N/m (Bruker) were used, and a borosilicate glass spherical ball of 20 μm in diameter (Novascan Tech) was attached using epoxy glue (Araldite). Cantilevers were calibrated using the thermal oscillation method and were tapped on the stromal regions, which closely connect to epithelial regions. Tissue indentation was conducted with the following settings: piezo displacement speed 2 μm s^−1^ and sampling rate 2000 Hz with a maximum force of 300 pN. Ten 30 μm by 30 μm AFM force maps were typically obtained at each stage, and each stiffness map was presented as a 5 × 5 raster series of indentations using the JPK software. Samples were assumed to be incompressible, a Poisson’s ratio of 0.5 was used, and Young’s modulus of each sample was calculated using a fit of the Hertz model.

### RNA extraction and real-time PCR

According to the manufacturer’s protocol, the RNA of organoids released from the hydrogels was extracted with an RNeasy Micro Kit (Qiagen). A cDNA Synthesis Kit (TaKaRa) was used to reverse transcribe RNA to cDNA. Real-time PCR was performed using the SYBR Green PCR Master Mix (TaKaRa) on an Applied Biosystems 7500 Fast Dx Real-Time PCR Instrument. The primers were designed by PrimerPremier5.0 and verified by Primer-BLAST and are shown in table S5. The relative expression of target genes was calculated by the 2^–ΔΔCt^ method. Glyceraldehyde-3-phosphate dehydrogenase was used as the loading control.

### Bulk mRNA sequencing and analysis

Organoids cultured in hydrogels with medium and high stiffness were collected. Total mRNA was extracted using TRIzol (Life Technologies) and then purified with an RNeasy Mini Kit (QIAGEN). RNA sequencing libraries were prepared using the TruSeq RNA Library Prep Kit v2 (Illumina), and the samples were sequenced on an Illumina HiSeq 2500 sequencer. Sequencing data were analyzed using the DESeq2 package (http://bioconductor.org/packages/release/bioc/html/DESeq2.html). Transcripts and genes were annotated according to the RefGene database (National Center for Biotechnology Information), and sample variability was visualized by principal components analysis. GO term enrichment analysis was performed using the goseq package (https://toppgene.cchmc.org/). The heatmaps were drawn using the R package pheatmap with the color range scaled to reflect the quantile distribution of the data.

### Statistical analysis

All experiments were performed at least three times. GraphPad Prism 6.0 was used to analyze the data. Statistical analysis was carried out using unpaired two-tailed Student’s *t* test when two groups were compared and by one-way analysis of variance (ANOVA) followed by Dunnett’s multiple comparison test (versus control group), or Tukey’s multiple comparisons test (versus every other group) was performed to compare multiple groups. The results are presented as means ± SEM, and *P* < 0.05 was considered statistically significant.
