# Supplementary material for: Varying mechanical forces drive sensory epithelium formation
Source: Sci Adv. 2023 Nov 3;9(44):eadf2664. doi: 10.1126/sciadv.adf2664 (PMC10624343; doi:10.1126/sciadv.adf2664)
Supplement: Supplementary file 1 — Figs. S1 to S8 Tables S1 to S5 [file sciadv.adf2664_sm.pdf]

Science Advances
AAAS

Supplementary Materials for

Varying mechanical forces drive sensory epithelium formation

Mingyu Xia *et al.*

Corresponding author: Wenyan Li, wenyan_li@fudan.edu.cn; Huawei Li, hwli@shmu.edu.cn; Yong He, yongqin@zju.edu.cn

Sci. Adv. **9**, eadf2664 (2023)
DOI: 10.1126/sciadv.adf2664

This PDF file includes:

Figs. S1 to S8
Tables S1 to S5

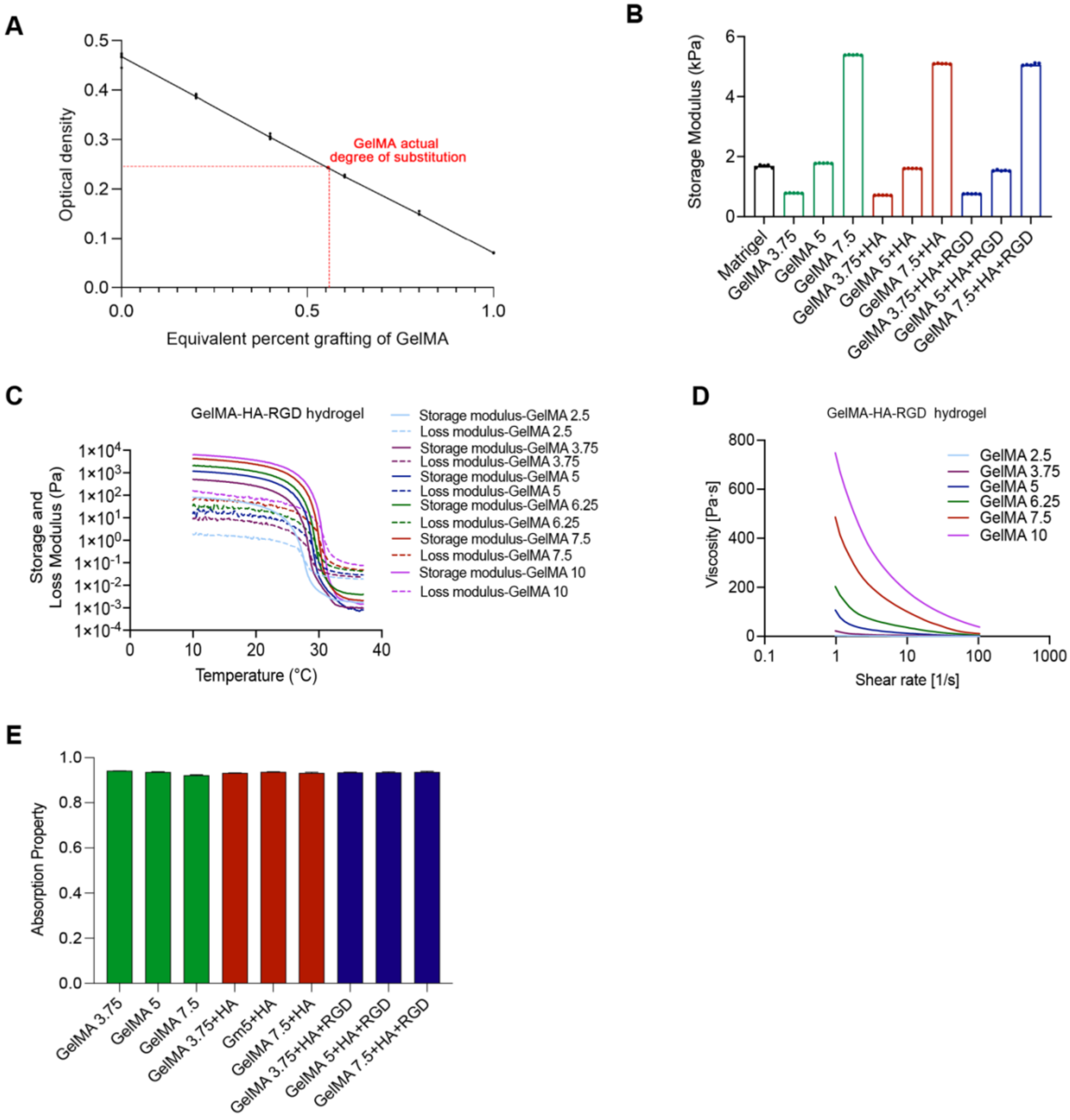

Fig. S1. Hybrid GelMA-HA-RGD hydrogel properties. (**A**) A fitted curve of the 2,4,6-trinitrobenzene-sulfonic acid assay showing GelMA degree of substitution. (**B**) Storage modulus of solidified Matrigel and crosslinked hydrogels. (**C**) Storage modulus and loss modulus of uncrosslinked hydrogels at different temperatures. (**D**) Rheological property of the different materials. (**E**) Water absorbency of the gels of the indicated parameters.

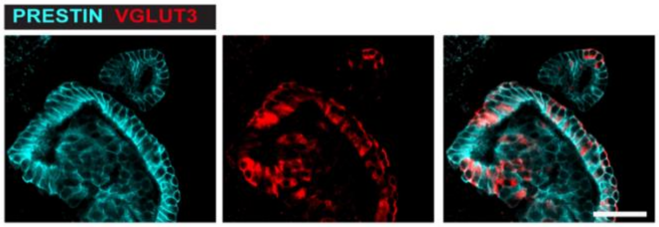

Fig. S2. Cells in organoids positively stained with inner hair cell and outer hair cell markers. Confocal images show VGLUT3 and PRESTIN staining of the organoids in 5 kPa hydrogels. Scale bar, 50 μm.

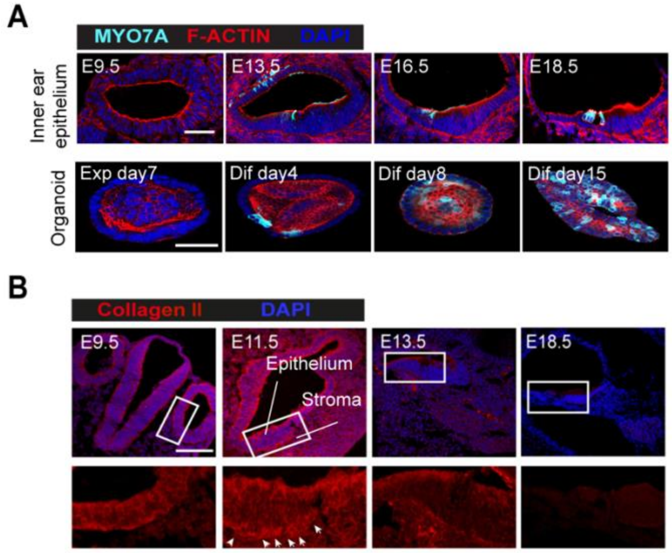

Fig. S3. ECM remodeling in developing inner ear epithelia. (**A**) Confocal images show MYO7A and F-actin staining of the developing inner ear epithelia and organoids. Scale bar, 50 μm. (**B**) Confocal images showing Collagen II staining of the developing inner ear epithelia. Scale bar, 100 μm. White arrows indicate the ends of the epithelium which is at the junction with the stroma.

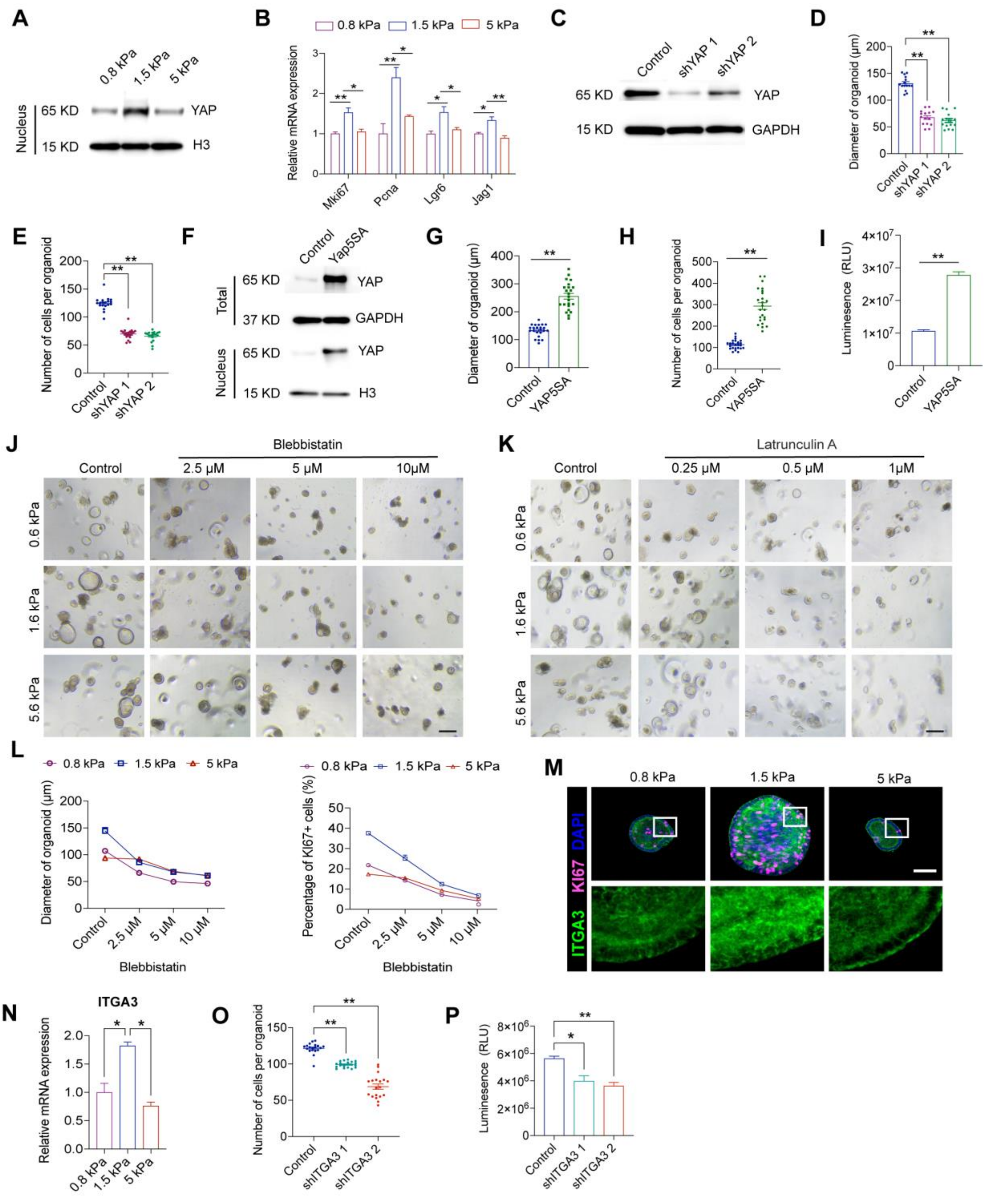

Fig. S4. Matrix Stiffness promotes cochlear organoid expansion that requires ITGA3-mediated F-actin cytoskeleton enrichment. (**A**) Western blot analysis showing the nuclear YAP expression. H3 was used as the loading control. (**B**) Real-time PCR analysis shows the relative expression of CPC marker genes and proliferation-related genes. The results were normalized to GAPDH in the same sample and then normalized to the control group (n = 3). (**C**) Western blot

analysis showing the YAP expression in hydrogels with 1.5 kPa. GAPDH was used as the loading control. (**D** and **E**) Quantifying the diameter of organoids and number of cells per organoid. 15-19 organoids at each condition. (**F**) Western blot analysis showing the total and nuclear YAP expression. GAPDH was used as the loading control for total protein. H3 was used as the loading control for nuclear protein. (**G-I**) Quantifying the diameter of organoids, number of cells per organoid, and cell viability. 23-24 organoids at each condition for (**G**) and (**H**). n = 3 independent experiments for (**I**). (**J** and **K**) Bright-field images showing CPCs cultured in indicated conditions. Scale bar, 100 μm. (**L**) Quantifying the diameter of organoids and the percentage of KI67+ cells per organoid. (M) Confocal images showing ITGA3 staining. Scale bar, 50 μm. (**N**) Real-time PCR analysis shows the relative expression of *ITGA3*. The results were normalized to GAPDH in the same sample and then normalized to the control group (n = 3). (**O** and **P**) Quantifying the diameter of organoids and cell viability. 19 organoids at each condition for (**O**). n = 3 independent experiments for (**P**). The data are presented as the mean ± SEM, *P < 0.05, **P < 0.01, ns, not significant. One-way ANOVA followed by Tukey's multiple comparisons test in (B), (**D**), (**E**), (**N**), (**O**), and (**P**). Unpaired Student's t-test in (**G-H**).

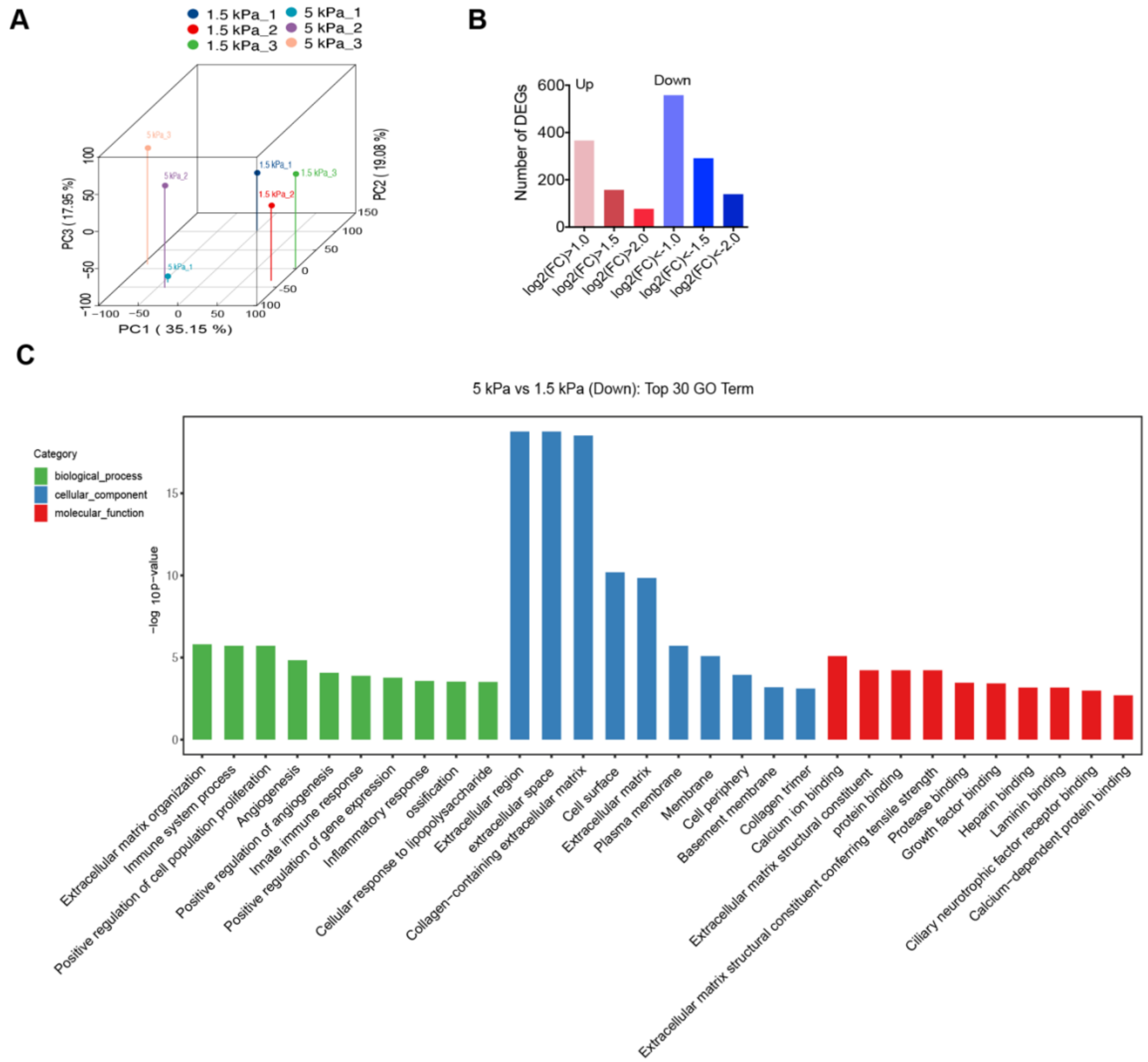

Fig. S5. RNA sequencing of the cochlear organoids. (**A**) Principal component analysis plot showing cochlear organoids cultured in 1.5 kPa and 5 kPa hydrogels. (**B**) The number of upregulated and downregulated DEGs with different fold changes (organoids in 5 kPa *vs.* 1.5 kPa). (**C**) Top 30 GO terms of the downregulated genes related to organoids in 5 kPa gels *vs.* 1.5 kPa gels.

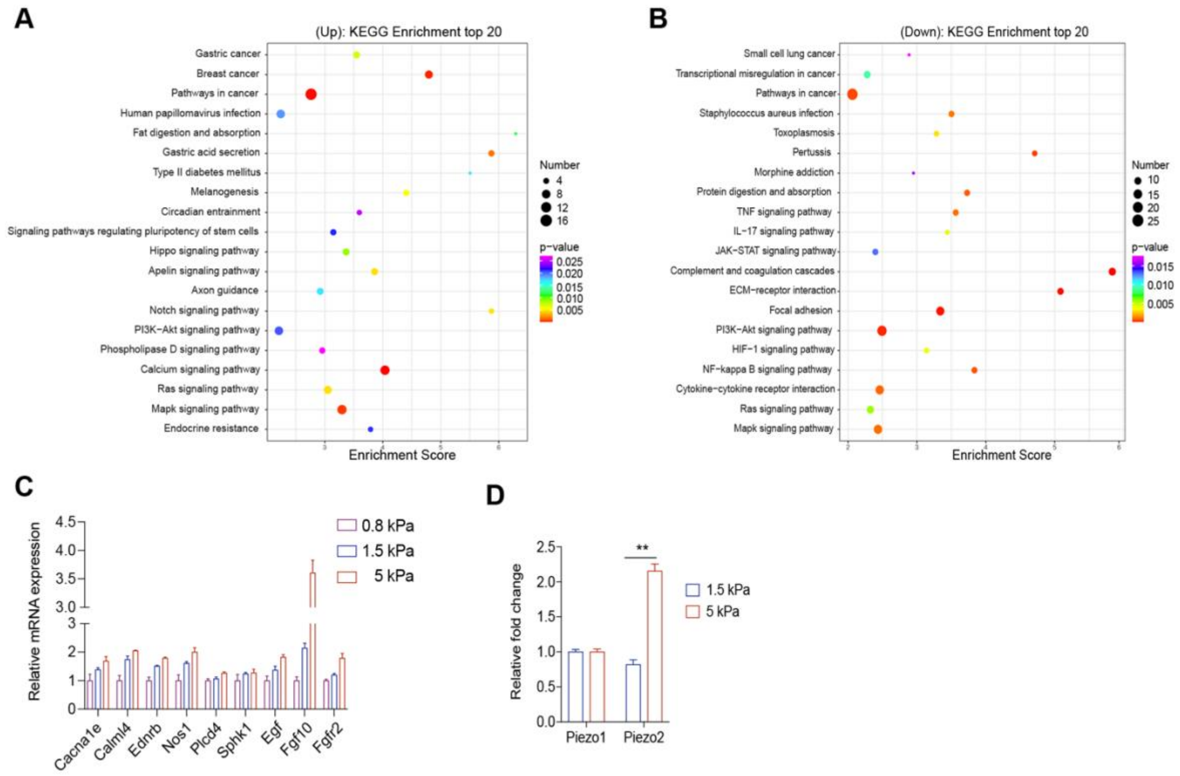

Fig. S6. KEGG pathway analysis of the cochlear organoids. (**A**) Top 20 enriched KEGG pathways of the upregulated genes related to organoids in 5 kPa gels *vs.* 1.5 kPa gels. (**B**) Top 20 enriched KEGG pathways of the downregulated genes related to organoids in 5 kPa gels *vs.* 1.5 kPa gels. (**C**) Real-time PCR verified organoids' calcium signaling pathway-related genes cultured in hydrogels with different stiffness. (**D**) Real-time PCR confirmed the focal adhesion-related genes of organoids cultured in hydrogels with different stiffness. Results were normalized to GAPDH in the same sample and then normalized to the control group (n = 3). (**E**) RNA sequencing data of the expression of *Piezo1* and *Piezo2* related to organoids in 5 kPa gels *vs.* 1.5 kPa gels.

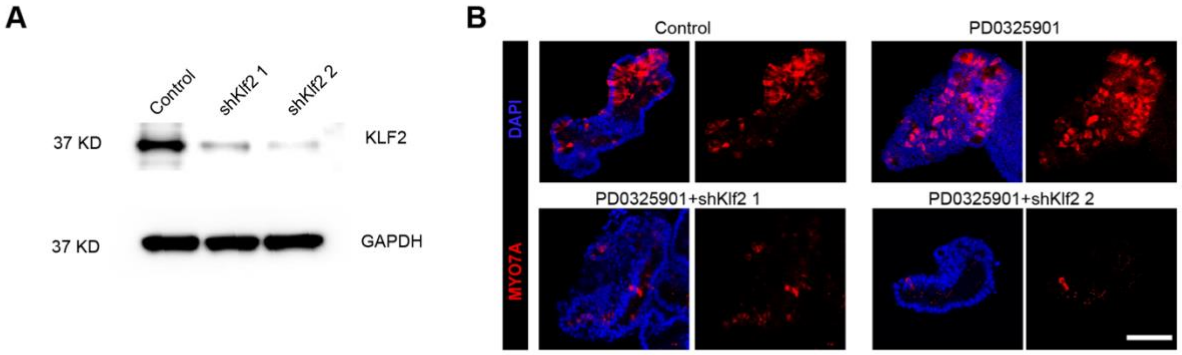

Fig. S7. KLF2 is essential for cochlear organoid differentiation promoted by matrix stiffness. **(A)** Western blot analysis showing the KLF2 expression in organoids after *KLF2* KD in hydrogels with 5 kPa. GAPDH was used as the loading control. (**B**) Confocal images showing MYO7A staining. Scale bar, 50 μm.

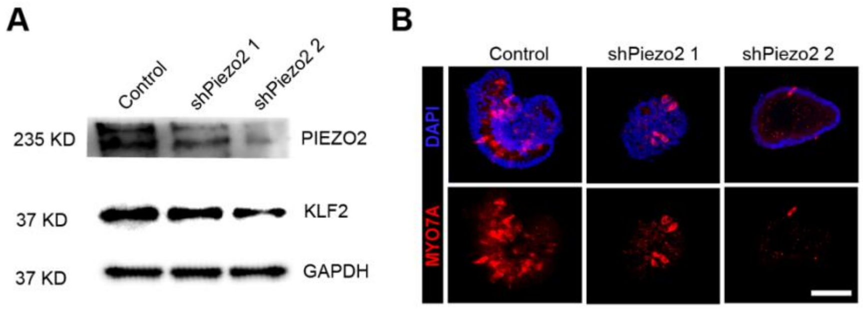

Fig. S8. PIEZO2 is essential for cochlear organoid differentiation promoted by matrix stiffness. (A) Western blot analysis showing the PIEZO2 and KLF2 expression in organoids after *Piezo2* KD in hydrogels with 5 kPa. GAPDH was used as the loading control. (**B**) Confocal images showing MYO7A staining. Scale bar, 50 μm.

Table S1: Animal models

Mouse models	Source	Identifier
Lgr5-EGFP-IRES-creERT2	Abcam	Stock #008875
Rosa26-tdTomato	Cell Signaling Technology	Stock #007914
Atoh1-EGFP	Obtained from J. Johnson (University of Texas Southwestern Medical Center, Dallas, TX, USA)	n/a
Atoh1-CreER	Obtained from S. Baker (St. Jude Children’s Research Hospital, Memphis, TN, USA)	n/a

Table S2: Primers for genotyping

Gene	Forward Primer	Reverse primer
Lgr5CreER WT	5’-CTGCTCTCTGCTCCCAGTCT -3’	5’ - ATACCCCATCCCTTT TGAGC -3’
Lgr5 CreER Mut	5’-CTGCTCTCTGCTCCCAGTCT -3’	5’ - GAACTTCAGGGTCAG CTTGC -3’
tdTomato WT	5’-AAGGGAGCTGCAGTGGAGTA-3’	5’-CCGAAAATCTGTGGGAAGTC-3’
tdTomato Mut	5’-CTGTTCCTGTACGGCATGG-3’	5’-GGCATTAAAGCAGCGTATCC-3’
Atoh1-EGFP WT	5’-GCGGTCTGGCAGTAAAAACTATC-3’	5’-GTGAAACAGCATTGCTGTCACTT-3’
Atoh1-EGFP Mut	5’ -AAGTTCATCTGCACCACCG-3’	5’ -TCCTTGAAGAAGATGGTGCG-3’
Atoh1 CreER WT	5’-CTAGGCCACAGAATTGAAAGATCT-3’	5’-GTAGGTGGAAATTCTAGCATCATCC-3’
Atoh1 CreER Mut	5’-GCGGTCTGGCAGTAAAAACTATC-3’	5’-GTGAAACAGCATTGCTGTCACTT-3’

Table S3: shRNA oligo sequences

Oligo name	Oligo Sequences
1. ShYAP Forward	5’–GATCCGCCAGTACTGATGCAGGTACTCTCGAGAGTACCTGCATCAGTACTG GCTTTTTT–3’
1. ShYAP Reverse	5’–AATTAAAAAAGCCAGTACTGATGCAGGTACTCTCGAGAGTACCTGCATCAG TACTGGCG–3’
2. ShYAP Forward	5’–GATCCGCTGATGAATTCTGCCTCAGGCTCGAGCCTGAGGCAGAATTCATCA GCTTTTTT–3’
2. ShYAP Reverse	5’–AATTAAAAAAGCTGATGAATTCTGCCTCAGGCTCGAGCCTGAGGCAGAATTC ATCAGCG–3’
1. ShKlf2 Forward	5’–CCGGCCTAAACAACGTGTTGGACTTCTCGAGAAGTCCAACACGTTGTTTAG GTTTTTTG–3’
1. ShKlf2 Reverse	5’–AATTCAAAAAACCTAAACAACGTGTTGGACTTCTCGAGAAGTCCAACACGT TGTTTAGG–3’
2. ShKlf2 Forward	5’–CCGGGCAAACAGACTGCTATTTATTCTCGAGAATAAATAGCAGTCTGTTTGC TTTTTTG–3’
2. ShKlf2 Reverse	5’–AATTCAAAAGCAAACAGACTGCTATTTATTCTCGAGAATAAATAGCAGTC TGTTTGC–3’
1. ShPiezo2 Forward	5’–CCGGGCATGATGCTGCCATCTTTCTCAAGAGAAAAGATGGCAGCATCATGCTT TTTTG–3’
1. ShPiezo2 Reverse	5’–AATTCAAAAAAGCATGATGCTGCCATCTTTTCTCTTGAGAAAGATGGCAGC ATCATGC–3’

2. ShPiezo2 Forward	5'–CAGGGCTTATCAATTGTGCCAAATTCAAGAGATTTGGCACAATTGATAAGC TTTTTTG–3'
2. ShPiezo2 Reverse	5'–AATTCAAAAAAGCTTATCAATTGTGCCAAATCTCTTGAATTTGGCACAATT GATAAGC–3'
1. ShITGA3 Forward	5'–CCGGGCCTCGCTCAGCTTAATGAATCTCGAGATTCATTAAGCTGAGCGAGG CTTTTTTG–3'
1. ShITGA3 Reverse	5'–AATTCAAAAAAGCCTCGCTCAGCTTAATGAATCTCGAGATTCATTAAGCTGAG CGAGGCC–3'
2. ShITGA3 Forward	5'–CCGGCCCTACTACTTCGAACGGAAACTCGAGTTTCCGTTCGAAGTAGTAGGG TTTTTTG–3'
2. ShITGA3 Reverse	5'–AATTCAAAAAACCCTACTACTTCGAACGGAAACTCGAGTTTCCGTTCGAAGTA GTAGGG–3'
ShControl Forward	5' – CCGGCCTAAGGTTAAGTCGCCCTCGCTCGAGCGAGGGCGACTTAACCTTAGG TTTTTTG – 3'
ShControl Reverse	5' – AATTCAAAAAACCTAAGGTTAAGTCGCCCTCGCTCGAGCGAGGGCGACTTAA CCTTAGG – 3'

Table S4. List of antibodies and reagents

**Antibodies**	**Supplier**	**Identifier**
Rabbit anti-KI67	Abcam	Cat# ab15580, RRID: AB_443209
Rabbit anti-GAPDH	Abcam	Cat# ab8245, RRID: AB_2107448
Rabbit anti-YAP	Cell Signaling Technology	Cat# 15117, RRID: AB_2798714
Rabbit anti-MYO7A	Proteus BioSciences	Cat# a25-6790, RRID: AB_2314838
Mouse anti-β-CATENIN	Santa Cruz Biotechnology	Cat# SC-7963, RRID: AB_626807
Goat anti-SOX2	Santa Cruz Biotechnology	Cat# SC17319, RRID: AB_661259
Mouse anti-POU4F3	Santa Cruz Biotechnology	Cat#SC-81980; RRID: AB_2167543
Anti-E-CADHERIN, Alexa Fluor 488	Thermo Fisher Scientific	Cat# 53-3249-82, RRID: AB_10671003
Rabbit Anti-ZO-1	Thermo Fisher Scientific	Cat# 61-7300, RRID: AB_138452
Rabbit Anti-MYO6	Thermo Fisher Scientific	Cat# PA5-110004, RRID: AB_2855415
Rabbit anti-MYO II	Cell Signaling Technology	Cat# 3403S, RRID: AB_2147297
Rabbit anti-ESPIN	Cell Signaling Technology	Cat# ab91509, RRID: AB_2049622
Rabbit anti-p-ERK1/2	Cell Signaling Technology	Cat# 4370t, RRID: AB_2315112
Rabbit anti-ERK1/2	Cell Signaling Technology	Cat# 4695T, RRID: AB_331646
Rabbit KLF2	LifeSpan BioSciences	Cat# LS-C30851-100, RRID: AB_903968)
Rabbit anti-Collagen II	Affinity Biosciences	Cat# AF0135, RRID: AB_2833318
Mouse anti-ITGA3	Proteintech	Cat# 66070-1-lg, RRID: AB_11042765
Mouse Anti-PARVALBUMIN	Sigma-Aldrich	Cat# P3088, RRID: AB_477329
**Chemicals and recombinant proteins**		
Gelatin	Sigma-Aldrich	924504
MA	Sigma-Aldrich	276685
HA	MACKLIN	H874944
RGD	EngineeringForLife	EFL-Rep-RGDfKAC
LAP (photoinitiator lithium phenyl-2,4,6-trimethyl-benzoylphosphinate)	EngineeringForLife	EFL-LAP
Matrigel	Corning	354230
Gelma Lysis Solution	EngineeringForLife	EFL-GM-LS-001
Cell recovery solution	Corning	354253
EGF	Peprotech	315-09
bFGF	Peprotech	450-33

IGF	Peprotech	250-19
CHIR99021	Sigma-Aldrich	SML1046
Y-27632	Sigma-Aldrich	Y0503
NAC	Sigma-Aldrich	A9165
PD0325901	Sigma-Aldrich	PZ0162
Latrunculin A	Sigma Aldrich	428021
Puromycin	Sigma Aldrich	540411
Blebbistatin	Selleck	E1249
LY411575	Selleck	S2714
F-actin Readyprobes	Thermo Fisher Scientific	R37112
EdU, Alexa Fluor 647	Thermo Fisher Scientific	C10635
DMEM/F-12, GlutaMAX	Thermo Fisher Scientific	10565042
DAPI	Thermo Fisher Scientific	D1306
N-2 Supplement	Thermo Fisher Scientific	A13707
B-27 Supplement	Thermo Fisher Scientific	17504044
Penicillin-Streptomycin	Thermo Fisher Scientific	15070063
Trypsin	Thermo Fisher Scientific	25200056
Trypsin neutralizer solution	Thermo Fisher Scientific	R002100
Fluo-4 AM	R&D System	273221-67-3
DMSO	Sigma Aldrich	472301
Optimal cutting temperature compound	Sakura Finetek	4583

Table S5：Primers for real-time PCR

Gene	Forward Primer	Reverse primer
Pcna	5’-TTTGAGGCACGCCTGATCC-3’	5’-GGAGACGTGAGACGAGTCCAT-3’
Myo6	5’-TGTTAAGGCAGGTTCCTTGAAG-3’	5’-ACACCAGCTACAACTCGAAAC-3’
Lgr6	5’-GAGGACGGCATCATGCTGTC-3’	5’-GCTCCGTGAGGTTGTTCATACT-3’
Jag1	5’-CCTCGGGTCAGTTTGAGCTG-3’	5’-CCTTGAGGCACACTTTGAAGTA-3’
Mki67	5’-ATCATTGACCGCTCCTTTAGGT-3’	5’-GCTCGCCTTGATGGTTCCT-3’
Myo7a	5’-CATCCGCCAGTACACCAACAA-3’	5’-TCCCCGCTGATAATACAGCAC-3’
Pvalb	5’-ATCAAGAAGGCGATAGGAGCC-3’	5’ -GGCCAGAAGCGTCTTTGTT-3’
Slc17a8	5’-AGAATGCCGTGGGAGACTC-3’	5’-CAGTCACAGATGTACCGCTTG-3’
Slc26a5	5’-GAAAGGCCCATCTTCAGTCATC-3’	5’-GCCACTTAGTGATAGGCAGGAAC-3’
Atoh1	5’-GAGTGGGCTGAGGTAAAAGAGT-3’	5’- GGTCGGTGCTATCCAGGAG-3’
Pou4f3	5’-ATGCGCCGAGTTTGTCTCC-3’	5’- GGGCTTGAACGGATGGTTCT-3’
ITGA3	5’-CCTCTTCGGCTACTCGGTC-3’	5’-CCGGTTGGTATAGTCATCACCC-3’
Cacna1e	5’-GATGGAGACTCGGACCAGAG-3’	5’-TGACCGTGAAACAGTTCTGCC-3’
Calml4	5’-TCTTCTGGTGTCCATGAGGTG-3’	5’-GAAGTCCAGCTCTCCGTTCTT-3’
Ednrb	5’-GTGGCTTCTTGGGGGTATGG-3’	5’-TCTTAGTGGGTGGCGTCATTA-3’
Nos1	5’-CTGGTGAAGGAACGGGTCAG-3’	5’-CCGATCATTGACGGCGAGAAT-3’
Plcd4	5’-GAAGGTTATGAAGTGTCCGATGT-3’	5’-AACTGCTTTGACAAGAGAATGGA-3’
Sphk1	5’-AAAATACTGAGAAACTCGGTCGG-3’	5’-GCATCGCTTCTTAAAGTCCAGA-3’
Egf	5’-AGCATCTCTCGGATTGACCCA-3’	5’-CCTGTCCCGTTAAGGAAAACTCT-3’
Fgf10	5’-TTTGGTGTCTTCGTTCCCTGT-3’	5’-TAGCTCCGCACATGCCTTC-3’
Klf2	5’-CTCAGCGAGCCTATCTTGCC-3’	5’-CACGTTGTTTAGGTCCTCATCC-3’
Fgfr2	5’-AATCTCCCAACCAGAAGCGTA-3’	5’-CTCCCCAATAAGCACTGTCCT-3’

GAPDH	5’-AGGTCGGTGTGAACGGATTTG-3’	5’-TGTAGACCATGTAGTTGAGGTCA-3’
